# Synthesis, antiproliferative and antitrypanosomal activities, and DNA binding of novel 6-amidino-2-arylbenzothiazoles

**DOI:** 10.1080/14756366.2021.1959572

**Published:** 2021-08-30

**Authors:** Livio Racané, Valentina Rep, Sandra Kraljević Pavelić, Petra Grbčić, Iva Zonjić, Marijana Radić Stojković, Martin C. Taylor, John M. Kelly, Silvana Raić-Malić

**Affiliations:** a Faculty of Textile Technology, Department of Applied Chemistry, University of Zagreb, Zagreb, Croatia; b Faculty of Chemical Engineering and Technology, Department of Organic Chemistry, University of Zagreb, Zagreb, Croatia; c Faculty of Health Studies, University of Rijeka, Rijeka, Croatia; d Division of Organic Chemistry and Biochemistry, Ruđer Bošković Institute, Zagreb, Croatia; e Department of Infection Biology, London School of Hygiene and Tropical Medicine, London, UK

**Keywords:** Benzothiazole, amidine, antiproliferative activity, *Trypanosoma brucei*, ctDNA binding

## Abstract

A series of 6-amidinobenzothiazoles, linked via phenoxymethylene or directly to the 1,2,3-triazole ring with a *p*-substituted phenyl or benzyl moiety, were synthesised and evaluated *in vitro* against four human tumour cell lines and the protozoan parasite *Trypanosoma brucei*. The influence of the type of amidino substituent and phenoxymethylene linker on antiproliferative and antitrypanosomal activities was observed, showing that the imidazoline moiety had a major impact on both activities. Benzothiazole imidazoline **14a**, which was directly connected to *N*-1-phenyl-1,2,3-triazole, had the most potent growth-inhibitory effect (IC_50_ = 0.25 µM) on colorectal adenocarcinoma (SW620), while benzothiazole imidazoline **11b**, containing a phenoxymethylene linker, exhibited the best antitrypanosomal potency (IC_90_ = 0.12 µM). DNA binding assays showed a non-covalent interaction of 6-amidinobenzothiazole ligands, indicating both minor groove binding and intercalation modes of DNA interaction. Our findings encourage further development of novel structurally related 6-amidino-2-arylbenzothiazoles to obtain more selective anticancer and anti-HAT agents.

## Graphical Abstract

**Figure:**
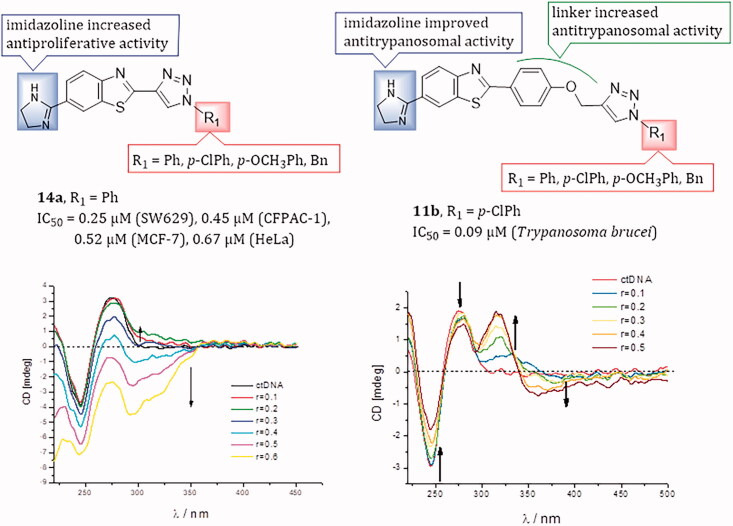

## Introduction

The benzothiazoles are constituents of bioactive heterocyclic compounds that exhibit a wide spectrum of biological activities^1–5^. Electron deficient, bivalent sulphur atoms in sulphur-containing heterocycles were found to participate in attractive nonbonding sulphur aromatic and sulphur halogen interactions that proved to enhance drug − target binding affinity^6^. Functionalization of the benzothiazole scaffold at the C-2 and C-6 positions has been a key determinant for their enhanced biological activity, mainly antiproliferative and antiparasitic^7–11^. Thus, the antiproliferative activity of amidino- and amino-substituted 2-phenylbenzothiazole derivatives strongly depended on the position of the substituent in the benzothiazole skeleton, as well as on the type of amidino unit^12–16^. Anticancer effects of 2-arylbenzothiazoles involved metabolic activation by cytochrome P450 to electrophilic reactive species, which generated DNA adducts in sensitive tumour cells^17^. Polyhydroxylated 2-phenylbenzothiazoles were developed as surrogates for the naturally occurring bioactive flavonoid and isoflavone^18^. Among C-2-arylbenzothiazoles, amino-substituted derivatives possessed excellent cytotoxicity in nanomolar concentrations against several breast cancer cell lines^19^^,^^20^. While the methylated analogue of C-2-arylbenzothiazole demonstrated superior *in vivo* efficacy, albeit, with metabolic instability, the fluorinated analogue exhibited enhanced stability with limited bioavailability^17^. The prodrug concept led to the development of the phortress compound, which had potent antitumor activity against human mammary tumour xenografts and is in clinical trials for the treatment of solid tumours^21^. 2-Piperazinyl benzothiazole derivatives were found to strongly inhibit the growth of hepatocellular, breast, and colorectal cancer cells^22^. Even though a number of structurally related benzothiazoles have been reported to exert antitumor effect, their mechanism is not fully evaluated, as a prelude to lead optimisation and clinical development. Some benzothiazole-based anticancer agents were found to target tyrosine kinase, topoisomerase, microtubule, cytochrome P450, heat shock protein 90 (Hsp90), epidermal growth factor receptor (EGFR) and apoptosis by activation of reactive oxygen species (ROS)^2^^,^^23–29^. Amidino benzothiazoles that exhibited strong antiproliferative activity also exerted good DNA binding affinity having both helix groove binding and DNA intercalation properties^30^. Some benzothiazole sulphonamides were found to have a crucial role in the inhibition of the metalloenzyme carbonic anhydrase (CA IX and XII) that is overexpressed in hypoxic tumours^1^^,^^31^^,^^32^.

Human African trypanosomiasis (HAT,) or sleeping sickness, is a neglected tropical disease (NTD) caused by *Trypanosoma brucei*, a protozoan parasite transmitted to humans through the bite of a blood-sucking tsetse fly^33^^,^^34^. The drugs currently used to treat HAT are not effective against all stages and subspecies of the parasite, so further clinical investigation is needed to develop new antitrypanosomal drugs. Some 2-benzylsulfanyl- and 2-arylbenzothiazole derivatives have been found to exhibit good trypanocidal activity at low concentrations^35^^,^^36^. Optimisation of anti-parasite activity, physicochemical parameters and ADME properties afforded the fluoro-substituted benzothiazole, with a 2-cyclopropanecarboxamide at position 2, which displayed promising *in vivo* efficacy^37^.

**Figure 1.**
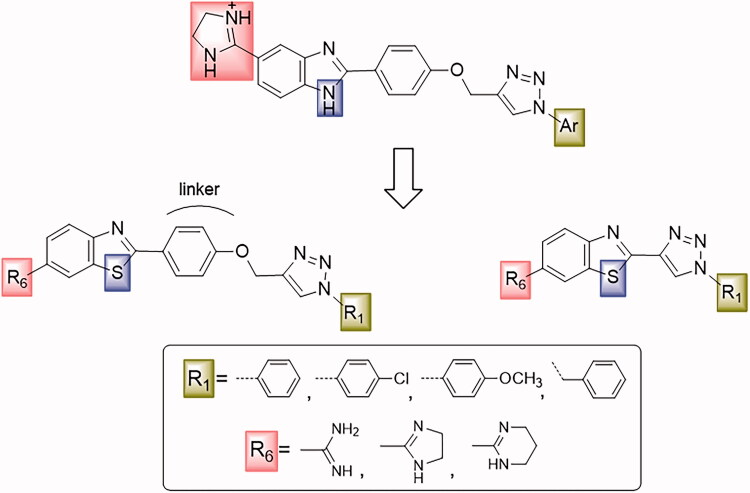
Design and synthesis of benzothiazoles with amidines as the hydrophilic end, and an aromatic unit at the N-1 position of the 1,2,3-triazole ring, as the hydrophobic end.

In continuation of our recent work on the development of aromatic benzimidazole amidines as antitrypanosomal^38–40^ and cytostatic agents^41^, we have now synthesised new chemical entities by the fusion of benzothiazole through a phenoxymethylene unit, or directly to 1,2,3-triazole ring with a *p*-substituted phenyl or benzyl subunit (Figure 1).

In this context, the influence of linkers and the type of amidino substituents of the benzothiazole derivatives **10a–10d**, **11a–11d**, **12a–12d**, **13a–13d**, **14a–14d**, **15a–15d** on their antiproliferative and antiprotozoal activity has been explored. Compounds with potent antiproliferative and antitrypanosomal activities were selected for further investigation of their DNA binding affinities by UV-Vis and CD spectroscopy, as well as thermal denaturation experiments.

## Materials and methods

### General

Melting points were determined by means of Original Kofler Mikroheitztisch apparatus (Reichert, Wien). ^1^H NMR and ^13^C NMR spectra were recorded with the Bruker Avance DPX-300 or Bruker AV-600 using TMS as an internal standard. Chemical shifts are reported in parts per million (ppm) relative to TMS. UPLC-MS spectra were recorded with Agilent 1290 Infiniti II/6120 Quadruple LC/MS spectrometers using electrospray ionisation (ESI). Elemental analyses for carbon, hydrogen, and nitrogen were performed on a Perkin-Elmer 2400 elemental analyser. Analyses are indicated as symbols of elements, and the analytical results obtained are within 0.4% of the theoretical value.

### Experimental procedures for the synthesis of compounds

Synthesis of main precursors for the synthesis of targeted benzothiazoles, namely 4-(prop-2-yn-1-yloxy)benzaldehyde (**2**)^41^, 4-[(1-phenyl-1*H*-1,2,3-triazol-4-yl)methoxy]benzaldehyde (**3a**)^41^, 4-{[1-(4-chlorophenyl)-1*H*-1,2,3-triazol-4-yl]methoxy}benzaldehyde (**3b**)^41^, 4-{[1-(4-methoxyphenyl)-1*H*-1,2,3-triazol-4-yl]methoxy}benzaldehyde (**3c**)^38^, 4-[(1-benzyl-1*H*-1,2,3-triazol-4-yl)methoxy]benzaldehyde (**3d**)^41^, (1-phenyl-1*H*-1,2,3-triazol-4-yl)methanol (**5a**)^42^, [1-(4-chlorophenyl)-1*H*-1,2,3-triazol-4-yl]methanol (**5b**)^41^, [1-(4-methoxyphenyl)-1*H*-1,2,3-triazol-4-yl]methanol (**5c**)^42^, (1-benzyl-1*H*-1,2,3-triazol-4-yl)methanol (**5d**)^43^, 1-phenyl-1*H*-1,2,3-triazole-4-carbaldehyde (**6a**)^44^, 1-(4-chlorophenyl)-1*H*-1,2,3-triazole-4-carbaldehyde (**6b**)^41^, 1-(4-methoxyphenyl)-1*H*-1,2,3-triazole-4-carbaldehyde (**6c**)^42^, 1-benzyl-1*H*-1,2,3-triazole-4-carbaldehyde (**6d**)^43^, 2-amino-5-amidiniumbenzenethiolate (**7**)^45^, 2-amino-5-(4,5-dihydro-1*H*-imidazol-3-ium-2-yl)benzenethiolate hydrate (**8**)^45^ and 2-amino-5-(3,4,5,6-tetrahydropyrimidin-1-ium-2-yl)benzenethiolate (**9**)^46^ was carried out according to the previously published experimental procedures.

### General method for preparation of compounds 10a–10d, 11a–11d, 12a–12d, 13a–13d, and 14a–14d

To a stirred solution of a corresponding 5-amidino-substituted-2-amino-benzenethiolate (**7–9**) (0.25 or 0.5 mmol) in glacial acetic acid (5 ml), a corresponding carbaldehyde **3a–3d** or **6a–6d** (0.25 or 0.5 mmol) was added under a nitrogen atmosphere and heated to reflux for 2–4 h. The reaction mixture was poured onto ice and made alkaline with 20% NaOH. The resulting free base was filtered, washed with water, and dried. The crude free base was converted into a methanesulfonate salt as described below.

#### 6-Amidinium-2-[(1-phenyl-1H-1,2,3-triazol-4-yl)methoxy]benzothiazole methanesulfonate (10a)

According to the above-mentioned general method, 4-[(1-phenyl-1*H-*1,2,3-triazol-4-yl)methoxy]benzaldehyde **3a** (70 mg, 0.25 mmol) and 2-amino-5-amidiniumbenzenethiolate **7** (42 mg, 0.25 mmol) were used and refluxed for 4 h, giving 78 mg (0.183 mmol) of crude free base. Afterwards, the obtained free base was suspended in ethanol (5 ml) followed by the addition of methanesulfonic acid (13 µl, 0.2 mmol) and stirring at room temperature for 2 h. The reaction mixture was cooled overnight, the resulting precipitate was filtered off and dried at 75 °C. Yield of pure compound **10a** as colourless solid was 60 mg (46.2%), mp = 272–275 °C. ^1^H NMR (300 MHz, DMSO-d6) *δ* = 9.35 (s, 2H, -C(N*H*_2_)_2_^+^), 8.99 (s, 2H, -C(N*H*_2_)_2_^+^), 8.95 (s, 1H, Ar-*H*), 8.63 (s, 1H, Ar-*H*), 8.21 (d, 1H, *J* = 8.5 Hz, Ar-*H*), 8.14 (d, 2H, *J* = 8.7 Hz, Ar-*H*), 7.93–7.90 (m, 3H, Ar-*H*), 7.64–7.49 (m, 3H, Ar-*H*), 7.32 (d, 2H, *J* = 8.6 Hz, Ar-*H*), 5.41 (s, 2H, -OC*H*_2_-), 2.35 (s, 3H, C*H*_3_SO_3_^–^). ^13^C NMR (75 MHz, DMSO-d6) *δ* = 171.2 (s), 165.3 (s), 161.1 (s), 156.7 (s), 143.3 (s), 136.5 (s), 134.5 (s), 129.9 (d, 2C), 129.4 (d, 2C), 128.8 (d), 126.3 (d), 125.3 (s), 124.6 (s), 123.2 (d), 123.1 (d), 122.5 (d), 120.2 (d, 2C), 115.7 (d, 2C), 61.34 (t). LC-MS (ESI) *m/z*: 427.3 (M + H^+^) calcd. for free base M = 426.13. Analysis calcd. for C_24_H_22_N_6_O_4_S_2_ (522.60): C, 55.16; H, 4.24; N, 16.08%; Found C, 54.98; H, 4.43; N, 16.01%.

#### 6-Amidinium-2-{[1-(4-chlorophenyl)-1H-1,2,3-triazol-4-yl]methoxy}benzothiazole methanesulfonate (10b)

According to the above-mentioned general method, 4-{[1-(4-chlorophenyl)-1*H*-1,2,3-triazol-4-yl]methoxy}benzaldehyde **3b** (79 mg, 0.25 mmol) and 2-amino-5-amidiniumbenzenethiolate **7** (42 mg, 0.25 mmol) were used and refluxed for 3 h, giving 103 mg (0.223 mmol) of crude free base. Afterwards, the obtained free base was suspended in ethanol (5 ml) followed by the addition of methanesulfonic acid (16 µl, 0.25 mmol) and stirring at room temperature for 2 h. The reaction mixture was cooled overnight, the resulting precipitate was filtered off, crystallised from ethanol and dried at 75 °C. Yield of pure compound **10b** as colourless solid was 51 mg (39.2%), mp = 276–279 °C. ^1^H NMR (300 MHz, DMSO-d6) *δ* = 9.23 (s, 4H, -C(N*H*_2_)_2_^+^), 9.04 (s, 1H, Ar-*H*), 8.63 (s, 1H, Ar-*H*), 8.22 (d, 1H, *J* = 8.8 Hz, Ar-*H*), 8.14 (d, 2H, *J* = 8.9 Hz, Ar-*H*), 7.98 (d, 2H, *J* = 8.8 Hz, Ar-*H*), 7.90 (d, 1H, *J* = 8.9 Hz, Ar-*H*), 7.70 (d, 2H, *J* = 8.5 Hz, Ar-*H*), 7.31 (d, 2H, *J* = 8.8 Hz, Ar-*H*), 5.40 (s, 2H, -OC*H*_2_-), 2.33 (s, 3H, C*H*_3_SO_3_^–^). ^13^C NMR (151 MHz, DMSO-d6) *δ* = 171.2 (s), 165.3 (s), 161.1 (s), 156.7 (s), 143.5 (s), 135.3 (s), 134.5 (s), 133.1 (s), 129.9 (d, 2C), 129.4 (d, 2C), 129.1 (d), 126.3 (d), 125.3 (s), 124.6 (s), 123.1 (d), 122.5 (d), 121.9 (d, 2C), 115.7 (d, 2C), 61.3 (t), 39.7 (q). LC-MS (ESI) *m/z*: 461.2 (M + H^+^) calcd. for free base M = 460.09. Analysis calcd. for C_24_H_21_ClN_6_O_4_S_2_ (557.04): C, 51.75; H, 3.80; N, 15.09%; Found C, 51.60; H, 3.98; N, 15.12%.

#### 6-Amidinium-2-{[1-(4-methoxyphenyl)-1H-1,2,3-triazol-4-yl]methoxy} benzothiazole methanesulfonate (10c)

According to the above-mentioned general method, 4-{[1-(4-methoxyphenyl)-1*H*-1,2,3-triazol-4-yl]methoxy}benzaldehyde **3c** (77 mg, 0.25 mmol) and 2-amino-5-amidiniumbenzenethiolate **7** (42 mg, 0.25 mmol) were used and refluxed for 3 h, giving 97 mg (0.212 mmol) of crude free base. Afterwards, the obtained free base was suspended in ethanol (5 ml) followed by the addition of methanesulfonic acid (16 µl, 0.25 mmol) and stirring at room temperature for 2 h. The reaction mixture was cooled overnight, the resulting precipitate was filtered off, crystallised from ethanol and dried at 75 °C. Yield of pure compound **10c** as colourless solid was 65 mg (45.5%), mp = 265–270 °C. ^1^H NMR (300 MHz, DMSO-d6) *δ* = 9.15 (s, 4H, -C(N*H*_2_)_2_^+^), 8.83 (s, 1H, Ar-*H*), 8.63 (s, 1H, Ar-*H*), 8.21 (d, 1H, *J* = 8.6 Hz, Ar-*H*), 8.13 (d, 2H, *J* = 8.8 Hz, Ar-*H*), 7.92 (d, 1H, *J* = 8.5 Hz, Ar-*H*), 7.81 (d, 2H, *J* = 8.9 Hz, Ar-*H*), 7.32 (d, 2H, *J* = 8.8 Hz, Ar-*H*), 7.15 (d, 2H, *J* = 8.9 Hz, Ar-*H*), 5.38 (s, 2H, -OC*H*_2_-), 3.85 (s, 3H, -OC*H*_3_), 2.34 (s, 3H, C*H*_3_SO_3_^–^). ^13^C NMR (151 MHz, DMSO-d6) *δ* = 171.2 (s), 165.3 (s), 161.1 (s), 159.3 (s), 156.7 (s), 143.1 (s), 134.5 (s), 129.9 (s), 129.4 (d, 2C), 126.3 (d), 125.3 (s), 124.6 (s), 123.1 (d), 123.0 (d), 122.5 (d), 121.8 (d, 2C), 115.7 (d, 2C), 114.9 (d, 2C), 61.4 (t), 55.6 (q), 39.7 (q). LC-MS (ESI) *m/z*: 457.2 (M + H^+^) calcd. for free base M = 456.14. Analysis calcd. for C_25_H_24_N_6_O_5_S_2_ × H_2_O (570.64): C, 52.62; H, 4.59; N, 14.73%; Found C, 52.71; H, 4.62; N, 14.68%.

#### 6-Amidinium-2-[(1-benzyl-1H-1,2,3-triazol-4-yl)methoxy]benzothiazole methanesulfonate (10d)

According to the above-mentioned general method, 4-[(1-benzyl-1*H-*1,2,3-triazol-4-yl)methoxy]benzaldehyde **3d** (148 mg, 0. 5 mmol) and 2-amino-5-amidiniumbenzenethiolate **7** (84 mg, 0.5 mmol) were used and refluxed for 2 h, giving 149 mg (0.277 mmol) of crude free base. Afterwards, the obtained free base was suspended in 2-propanole (10 ml) followed by the addition of methanesulfonic acid (20 µl, 0.31 mmol) and stirring at room temperature for 2 h. The reaction mixture was cooled overnight, the resulting precipitate was filtered off, crystallised from ethanol and dried at 75 °C. Yield of pure compound **10d** as pale yellow solid was 76 mg (28.4%), mp = 253–257 °C. ^1^H NMR (300 MHz, DMSO-d6) *δ* = 9.40 (s, 2H, -C(N*H*_2_)_2_^+^), 9.02 (s, 2H, -C(N*H*_2_)_2_^+^), 8.62 (d, 1H, *J* = 1.3 Hz, Ar-*H*), 8.34 (s, 1H, Ar-*H*), 8.22 (d, 1H, *J* = 8.6 Hz, Ar-*H*), 8.11 (d, 2H, *J* = 8.7 Hz, Ar-*H*), 7.89 (dd, 1H*, J* = 1.6 Hz, *J* = 8.7 Hz, Ar-*H*), 7.46–7.30 (m, 5H, Ar-*H*), 7.26 (d, 2H, *J* = 8.8 Hz, Ar-*H*), 5.63 (s, 2H, -C*H*_2_-), 5.28 (s, 2H, -OC*H*_2_-), 2.32 (s, 3H, C*H*_3_SO_3_^–^). ^13^C NMR (75 MHz, DMSO-d6) *δ* = 171.2 (s), 165.3 (s), 161.2 (s), 156.7 (s), 142.5 (s), 136.0 (s), 134.5 (s), 129.4 (d, 2C), 128.8 (d, 2C), 128.2 (d), 128.0 (d, 2C), 126.3 (d), 125.2 (s), 124.9 (d), 124.6 (s), 123.2 (d), 122.5 (d), 115.7 (d, 2C), 61.4 (t), 52.9 (t). LC-MS (ESI) *m/z*: 441.2 (M + H^+^) calcd. for free base M = 440.14. Analysis calcd. for C_25_H_24_N_6_O_4_S_2_ (536.63): C, 55.95; H, 4.51; N, 15.66%; Found C, 56.08; H, 4.36; N, 15.57%.

#### 6-(4,5-Dihydro-1H-imidazol-3-ium-2-yl)-2-[(1-phenyl-1H-1,2,3-triazol-4-yl)methoxy] benzothiazole methanesulfonate (11a)

According to the above-mentioned general method, 4-[(1-phenyl-1*H-*1,2,3-triazol-4-yl)methoxy]benzaldehyde **3a** (70 mg, 0.25 mmol) and 2-amino-5-(4,5-dihydro-1*H*-imidazol-3-ium-2-yl)benzenethiolate hydrate **8** (53 mg, 0.25 mmol) were used and refluxed for 2 h, giving 88 mg (0.195 mmol) of crude free base. Afterwards, the obtained free base was suspended in ethanol (5 ml) followed by the addition of methanesulfonic acid (15 µl, 0.23 mmol) and stirring at room temperature for 2 h. The reaction mixture was cooled overnight, the resulting precipitate was filtered off and dried at 75 °C. Yield of pure compound **11a** as colourless solid was 74 mg (54%), mp = 270–274 °C. ^1^H NMR (600 MHz, DMSO-d6) *δ* = 10.56 (s, 2H, -C(N*H*-)_2_^+^), 9.01 (s, 1H, Ar-*H*), 8.73 (s, 1H, Ar-*H*), 8.33–7.85 (m, 6H, Ar-*H*), 7.69–7.25 (m, 5H, Ar-*H*), 5.40 (s, 2H, -OC*H*_2_-), 4.06 (s, 4H, -C*H*_2_C*H*_2_-), 2.33 (s, 3H, C*H*_3_SO_3_^–^). ^13^C NMR (75 MHz, DMSO-d6) *δ* = 171.8 (s), 164.8 (s), 161.2 (s), 157.1 (s), 143.3 (s), 136.5 (s), 134.8 (s), 129.9 (d, 2C), 129.5 (d, 2C), 128.8 (d), 126.4 (d), 125.2 (s), 123.4 (d), 123.1 (d), 122.9 (d), 120.2 (d, 2C), 118.4 (s), 115.7 (d, 2C), 61.3 (t). LC-MS (ESI) *m/z*: 455.3 (M + H^+^) calcd. for free base M = 452.14. Analysis calcd. for C_26_H_24_N_6_O_4_S_2_ (548.64): C, 56.92; H, 4.41; N, 15.32%; Found C, 56.78; H, 4.37; N, 15.37%.

#### 6-(4,5-Dihydro-1H-imidazol-3-ium-2-yl)-2-{[1-(4-chlorophenyl)-1H-1,2,3-triazol-4-yl]methoxy}benzothiazole methanesulfonate (11b)

According to the above-mentioned general method, 4-{[1-(4-chlorophenyl)-1*H*-1,2,3-triazol-4-yl]methoxy}benzaldehyde **3b** (79 mg, 0.25 mmol) and 2-amino-5-(4,5-dihydro-1*H*-imidazol-3-ium-2-yl)benzenethiolate hydrate **8** (53 mg, 0.25 mmol) were used and refluxed for 2 h, giving 110 mg (0.225 mmol) of crude free base. Afterwards, the obtained free base was suspended in ethanol (10 ml) followed by the addition of methanesulfonic acid (16 µl, 0.25 mmol) and stirring at room temperature for 2 h. The reaction mixture was cooled overnight, the resulting precipitate was filtered off and dried at 75 °C. Yield of pure compound **11b** as colourless solid was 93 mg (64.1%), mp = 263–266 °C. ^1^H NMR (600 MHz, DMSO-d6) *δ* = 10.56 (s, 2H, -C(N*H*-)_2_^+^), 9.04 (s, 1H, Ar-*H*), 8.72 (s, 1H, Ar-*H*), 8.26 (d, *J* = 8.2 Hz, 1H, Ar-*H*), 8.14 (m, 2H, Ar-*H*), 8.05–7.93 (m, 3H, Ar-*H*), 7.70 (m, 2H, Ar-*H*), 7.32 (m, 2H, Ar-*H*), 5.40 (s, 2H, -OC*H*_2_-), 4.06 (s, 4H, -C*H*_2_C*H*_2_-), 2.32 (s, 3H, C*H*_3_SO_3_^–^). ^13^C NMR (151 MHz, DMSO-d6) *δ* = 171.8 (s), 164.9 (s), 161.2 (s), 157.1 (s), 143.5 (s), 135.3 (s), 134.8 (s), 133.1 (s), 129.9 (d, 2C), 129.5 (d, 2C), 126.4 (d), 125.2 (s), 123.4 (d), 123.2 (d), 123.0 (d), 121.9 (d, 2C), 118.4 (s), 115.7 (d, 2C), 61.31 (t), 44.5 (t, 2C), 39.7 (q). LC-MS (ESI) *m/z*: 487.2 (M + H^+^) calcd. for free base M = 486.10. Analysis calcd. for C_26_H_23_ClN_6_O_4_S_2_ (583.08): C, 53.56; H, 3.98; N, 14.41%; Found C, 53.43; H, 3.93; N, 14.34%.

#### 6-(4,5-Dihydro-1H-imidazol-3-ium-2-yl)-2-{[1-(4-methoxyphenyl)-1H-1,2,3-triazol-4-yl]methoxy}benzothiazole methanesulfonate (11c)

According to the above-mentioned general method, 4-{[1-(4-methoxyphenyl)-1*H*-1,2,3-triazol-4-yl]methoxy}benzaldehyde **3c** (77 mg, 0.25 mmol) and 2-amino-5-(4,5-dihydro-1*H*-imidazol-3-ium-2-yl)benzenethiolate hydrate **8** (53 mg, 0.25 mmol) were used and refluxed for 3 h, giving 101 mg (0.209 mmol) of crude free base. Afterwards, the obtained free base was suspended in ethanol (5 ml) followed by the addition of methanesulfonic acid (15 µl, 0.23 mmol) and stirring at room temperature for 2 h. The reaction mixture was cooled overnight, the resulting precipitate was filtered off and dried at 75 °C. Yield of pure compound **11c** as colourless solid was 82 mg (45.5%), mp = 253–257 °C. ^1^H NMR (600 MHz, DMSO-d6) *δ* = 10.55 (s, 2H, -C(N*H*-)_2_^+^), 8.89 (s, 1H, Ar-*H*), 8.72 (s, 1H, Ar-*H*), 8.26 (d, *J* = 8.2 Hz, 1H, Ar-*H*), 8.14 (d, 2H, *J* = 7.3 Hz, Ar-*H*), 8.01 (d, 1H, *J* = 8.3 Hz, Ar-*H*), 7.82 (d, 2H, *J* = 7.2 Hz, Ar-*H*), 7.32 (d, 2H, *J* = 7.3 Hz, Ar-*H*), 7.15 (d, 2H, *J* = 7.4 Hz, Ar-*H*), 5.38 (s, 2H, -OC*H*_2_-), 4.06 (s, 4H, -C*H*_2_C*H*_2_-), 3.84 (s, 3H, -OC*H*_3_), 2.31 (s, 3H, C*H*_3_SO_3_^–^). ^13^C NMR (75 MHz, DMSO-d6) *δ* = 171.8 (s), 164.8 (s), 161.3 (s), 159.3 (s), 157.1 (s), 143.1 (s), 134.8 (s), 129.9 (s), 129.5 (d, 2C), 126.4 (d), 125.1 (s), 123.4 (d), 123.1 (d), 122.9 (d), 121.8 (d, 2C), 118.4 (s), 115.7 (d, 2C), 114.9 (d, 2C), 61.4 (t), 55.6 (q), 44.6 (t, 2C). LC-MS (ESI) *m/z*: 483.3 (M + H^+^) calcd. for free base M = 482.15. Analysis calcd. for C_27_H_26_N_6_O_5_S_2_ (578.66): C, 56.04; H, 4.53; N, 14.52%; Found C, 56.18; H, 4.57; N, 14.51%.

#### 6-(4,5-Dihydro-1H-imidazol-3-ium-2-yl)-2[(1-benzyl-1H-1,2,3-triazol-4-yl)methoxy]benzothiazole methanesulfonate (11d)

According to the above-mentioned general method, 4-[(1-benzyl-1*H-*1,2,3-triazol-4-yl)methoxy]benzaldehyde **3d** (74 mg, 0.25 mmol) and 2-amino-5-(4,5-dihydro-1*H*-imidazol-3-ium-2-yl)benzenethiolate hydrate **8** (53 mg, 0.25 mmol) were used and refluxed for 2 h, giving 88 mg (0.182 mmol) of crude free base. Afterwards, the obtained free base was suspended in 2-propanol (5 ml) followed by the addition of methanesulfonic acid (13 µl, 0.20 mmol) and stirring at room temperature for 2 h. The reaction mixture was cooled overnight, the resulting precipitate was filtered off and dried at 75 °C. Yield of pure compound **11d** as colourless solid was 65 mg (46.1%), mp = 227–232 °C. ^1^H NMR (600 MHz, DMSO-d6) *δ* = 10.55 (s, 2H, -C(N*H*-)_2_^+^), 8.71 (s, 1H, Ar-*H*), 8.34 (s, 1H, Ar-*H*), 8.26 (d, 1H, *J* = 8.3 Hz, Ar-*H*), 8.12 (d, 2H, *J* = 7.4 Hz, Ar-*H*), 8.01 (d, 1H, *J* = 8.4 Hz, Ar-*H*), 7.46–7.30 (m, 5H, Ar-*H*), 7.27 (d, 2H, *J* = 7.5 Hz, Ar-*H*), 5.63 (s, 2H,-C*H*_2_-), 5.28 (s, 2H, -OC*H*_2_-), 4.06 (s, 4H, -C*H*_2_C*H*_2_-), 2.31 (s, 3H, C*H*_3_SO_3_^–^). ^13^C NMR (75 MHz, DMSO-d6) *δ* = 171.6 (s), 164.7 (s), 161.2 (s), 157.0 (s), 142.3 (s), 135.7 (s), 134.7 (s), 129.2 (d, 2C), 128.5 (d), 128.0 (d), 127.7 (d), 126.2 (d), 125.0 (s), 124.6 (d), 123.2 (d), 122.7 (d), 118.2 (s), 115.6 (d,2C), 61.4 (t), 52.7 (t), 44.4 (t, 2C), 39.6 (q). LC-MS (ESI) *m/z*: 467.3 (M + H^+^) calcd. for free base M = 466.16. Analysis calcd. for C_27_H_26_N_6_O_4_S_2_ (562.66): C, 57.63; H, 4.66; N, 14.94%; Found C, 57.47; H, 4.76; N, 15.01%.

#### 6-(3,4,5,6-Tetrahydropyrimidin-1-ium-2-yl)-2-[(1-phenyl-1H-1,2,3-triazol-4-yl)methoxy] benzothiazole methanesulfonate (12a)

According to the above-mentioned general method, 4-[(1-phenyl-1*H-*1,2,3-triazol-4-yl)methoxy]benzaldehyde **3a** (70 mg, 0.25 mmol) and 2-amino-5-(3,4,5,6-tetrahydropyrimidin-1-ium-2-yl)benzenethiolate **9** (52 mg, 0.25 mmol) were used and refluxed for 4 h, giving 75 mg (0.160 mmol) of crude free base. Afterwards, the obtained free base was suspended in 2-propanol (5 ml) followed by the addition of methanesulfonic acid (12 µl, 0.18 mmol) and stirring at room temperature for 2 h. The reaction mixture was cooled overnight, the resulting precipitate was filtered off and dried at 75 °C. Yield of pure compound **12a** as colourless solid was 64 mg (44.1%), mp = 249–254 °C. ^1^H NMR (300 MHz, DMSO-d6) *δ* = 9.95 (s, 2H, -C(N*H*-)_2_^+^), 8.95 (s, 1H, Ar-*H*), 8.52 (s, 1H, Ar-*H*), 8.22 (d, 1H, *J* = 8.7 Hz, Ar-*H*), 8.13 (d, 2H, *J* = 8.3 Hz, Ar-*H*), 7.92 (d, 2H, *J* = 7.7 Hz, Ar-*H*), 7.82 (d, 1H, *J* = 8.5 Hz, Ar-*H*), 7.69–7.48 (m, 3H, Ar-*H*), 7.32 (d, 2H, *J* = 8.5 Hz, Ar-*H*), 5.40 (s, 2H, -OC*H*_2_-), 3.55 (m, 4H, -C*H*_2_CH_2_C*H*_2_-), 2.30 (s, 3H, C*H*_3_SO_3_^–^), 2.04 (m, 2H, -CH_2_C*H*_2_CH_2_-). ^13^C NMR (151 MHz, DMSO-d6) *δ* = 170.7 (s), 161.0 (s), 159.0 (s), 156.2 (s), 143.2 (s), 136.4 (s), 134.4 (s), 129.7 (d, 2C), 129.2 (d, 2C), 128.6 (d), 125.6 (d), 125.2 (s), 124.8 (s), 122.8 (d), 122.5 (d), 122.2 (d), 120.0 (d, 2C), 115.6 (d, 2C), 61.3 (t), 39.64 (q), 38.8 (t, 2C), 17.6 (t). LC-MS (ESI) *m/z*: 467.3 (M + H^+^) calcd. for free base M = 466.16. Analysis calcd. for C_27_H_26_N_6_O_4_S_2_ × H_2_O (580.68): C, 55.85; H, 4.86; N, 14.47%; Found C, 56.09; H, 4.69; N, 14.58%.

#### 6-(3,4,5,6-Tetrahydropyrimidin-1-ium-2-yl)-2-[(1-(4-Chlorophenyl)-1H-1,2,3-triazol-4-yl]methoxy)benzothiazole methanesulfonate (12b)

According to the above-mentioned general method, 4-{[1-(4-chlorophenyl)-1*H*-1,2,3-triazol-4-yl]methoxy}benzaldehyde **3b** (79 mg, 0.25 mmol) and 2-amino-5-(3,4,5,6-tetrahydropyrimidin-1-ium-2-yl)benzenethiolate **9** (52 mg, 0.25 mmol) were used and refluxed for 3 h, giving 109 mg (0.218 mmol) of crude free base. Afterwards, the obtained free base was suspended in 2-propanol (10 ml) followed by the addition of methanesulfonic acid (15 µl, 0.23 mmol) and stirring at room temperature for 2 h. The reaction mixture was cooled overnight, the resulting precipitate was filtered off and dried at 75 °C. Yield of pure compound **12b** as colourless solid was 67 mg (45.0%), mp = 220–224 °C. ^1^H NMR (300 MHz, DMSO-d6) *δ* = 9.93 (s, 2H, -C(N*H*-)_2_^+^), 8.95 (s, 1H, Ar-*H*), 8.51 (s, 1H, Ar-*H*), 8.21 (d, 1H, *J* = 8.4 Hz, Ar-*H*), 8.13 (d, 2H, *J* = 8.6 Hz, Ar-*H*), 7.96 (d, 2H, *J* = 8.7 Hz, Ar-*H*), 7.82 (d, 1H, *J* = 7.2 Hz, Ar-*H*), 7.68 (d, 2H, *J* = 8.6 Hz, Ar-*H*), 7.31 (d, 2H, *J* = 8.6 Hz, Ar-*H*), 5.41 (s, 2H, -OC*H*_2_-), 3.55 (t, *J* = 5.3 Hz, 4H, -C*H*_2_CH_2_C*H*_2_-), 2.31 (s, 3H, C*H*_3_SO_3_^–^), 2.04 (m, 2H, -CH_2_C*H*_2_CH_2_-). ^13^C NMR (151 MHz, DMSO-d6) *δ* = 170.7 (s), 160.9 (s), 159.0 (s), 156.2 (s), 143.4 (s), 135.2 (s), 134.4 (s), 132.9 (s), 129.6 (d, 2C), 129.2 (d, 2C), 125.6 (d), 125.3 (s), 124.8 (s), 122.9 (d), 122.5 (d), 122.2 (d), 121.7 (d, 2C), 115.6 (d, 2C), 61.3 (t), 39.6 (q), 38.8 (t, 2C), 17.6 (t). LC-MS (ESI) *m/z*: 501.3 (M + H^+^) calcd. for free base M = 500.12. Analysis calcd. for C_27_H_25_ClN_6_O_4_S_2_ (583.08): C, 54.31; H, 4.22; N, 14.07%; Found C, 54.61; H, 3.99; N, 14.04%.

#### 6-(3,4,5,6-Tetrahydropyrimidin-1-ium-2-yl)-2-{[1-(4-methoxyphenyl)-1H-1,2,3-triazol-4-yl]methoxy}benzothiazole methanesulfonate (12c)

According to the above-mentioned general method, 4-{[1-(4-methoxyphenyl)-1*H*-1,2,3-triazol-4-yl]methoxy}benzaldehyde **3c** (77 mg, 0.25 mmol) and 2-amino-5-(3,4,5,6-tetrahydropyrimidin-1-ium-2-yl)benzenethiolate **9** (52 mg, 0.25 mmol) were used and refluxed for 2 h, giving 101 mg (0.203 mmol) of crude free base. Afterwards, the obtained free base was suspended in ethanol (5 ml) followed by the addition of methanesulfonic acid (15 µl, 0.23 mmol) and stirring at room temperature for 2 h. The reaction mixture was cooled overnight, diethyl-ether was added and the resulting precipitate was filtered off, and dried at 75 °C. Yield of pure compound **12c** as colourless solid was 60 mg (39.2%), mp = 259–264 °C. ^1^H NMR (300 MHz, DMSO-d6) *δ* = 9.96 (s, 2H, -C(N*H*-)_2_^+^), 8.84 (s, 1H, Ar-*H*), 8.52 (d, 1H, *J* = 1.5 Hz, Ar-*H*), 8.21 (d, 1H, *J* = 8.6 Hz, Ar-*H*), 8.13 (d, 2H, *J* = 8.8 Hz, Ar-*H*), 7.84–7.80 (m, 3H), 7.31 (d, 2H, *J* = 8.8 Hz, Ar-*H*), 7.15 (d, 2H, *J* = 9.0 Hz, Ar-*H*), 5.38 (s, 2H, -OC*H*_2_-), 3.85 (s, 3H, -OC*H*_3_), 3.55 (t, 4H, *J* = 5.6 Hz, -C*H*_2_CH_2_C*H*_2_-), 2.31 (s, 3H, C*H*_3_SO_3_^–^), 2.04 (m, 2H, -CH_2_C*H*_2_CH_2_-). ^13^C NMR (151 MHz, DMSO-d6) *δ* = 170.8 (s), 161.0 (s), 159.3 (s), 159.1 (s), 156.2 (s), 143.0 (s), 134.5 (s), 129.8 (s), 129.2 (d, 2C), 125.6 (d), 125.2 (s), 124.9 (s), 122.8 (d), 122.5 (d), 122.3 (d), 121.7 (d, 2C), 115.6 (d, 2C), 114.8 (d, 2C), 61.3 (t), 55.4 (q), 39.6 (q), 38.8 (t, 2C), 17.6 (t). LC-MS (ESI) *m/z*: 497.3 (M + H^+^) calcd. for free base M = 496.17. Analysis calcd. for C_28_H_28_N_6_O_5_S_2_ × H_2_O (610.70): C, 55.07; H, 4.95; N, 13.76%; Found C, 54.91; H, 4.99; N, 13.87%.

#### 6-(3,4,5,6-Tetrahydropyrimidin-1-ium-2-yl)-2-[(1-benzyl-1H-1,2,3-triazol-4-yl)methoxy]benzothiazole methanesulfonate (12d)

According to the above-mentioned general method, 4-[(1-benzyl-1*H-*1,2,3-triazol-4-yl)methoxy]benzaldehyde **3d** (148 mg, 0.5 mmol) and 2-amino-5-(3,4,5,6-tetrahydropyrimidin-1-ium-2-yl)benzenethiolate **9** (104 mg, 0.5 mmol) were used and refluxed for 2 h, giving 188 mg (0.391 mmol) of crude free base. Afterwards, the obtained free base was suspended in 2-propanol (5 ml) followed by the addition of methanesulfonic acid (28 µl, 0.43 mmol) and stirring at room temperature for 2 h. To the reaction mixture diethyl-ether was added, the resulting precipitates was filtered off, crystalised from 2-propanole and dried at 75 °C. Yield of pure compound **12d** as colourless solid was 47 mg (15.8%), mp = 197–199 °C. ^1^H NMR (300 MHz, DMSO-d6) *δ* = 10.01 (s, 2H, -C(N*H*-)_2_^+^), 8.52 (d, 1H, *J* = 1.3 Hz, Ar-*H*), 8.34 (s, 1H, Ar-*H*), 8.22 (d, 1H, *J* = 8.5 Hz, Ar-*H*), 8.11 (d, 2H, *J* = 8.7 Hz, Ar-*H*), 7.80 (dd, 1H, *J* = 1.5 Hz, *J* = 8.6 Hz, Ar-*H*), 7.42–7.32 (m, 5H, Ar-*H*), 7.26 (d, 2H, *J* = 8.8 Hz, Ar-*H*), 5.63 (s, 2H, -C*H*_2_-), 5.28 (s, 2H, -OC*H*_2_-), 3.53 (t, *J* = 5.4 Hz, 4H, -C*H*_2_CH_2_C*H*_2_-), 2.29 (s, 3H, C*H*_3_SO_3_^–^), 2.01 (m, 2H, -CH_2_C*H*_2_CH_2_-). ^13^C NMR (75 MHz, DMSO-d6) *δ* = 170.9 (s), 161.2 (s), 159.1 (s), 156.3 (s), 142.5 (s), 136.0 (s), 134.6 (s), 129.3 (d, 2C), 128.8 (d, 2C), 128.2 (d), 128.0 (d, 2C), 125.9 (d), 125.2 (s), 125.0 (s), 124.9 (d), 122.7 (d), 122.5 (d), 115.7 (d, 2C), 61.4 (t), 52.8 (t), 39.7 (q), 38.9 (t, 2C), 17.7 (t). LC-MS (ESI) *m/z*: 481.3 (M + H^+^) calcd. for free base M = 480.17. Analysis calcd. for C_28_H_28_N_6_O_4_S_2_ × H_2_O (594.70): C, 56.55; H, 5.08; N, 14.13%; Found C, 56.78; H, 5.26; N, 14.02%.

#### 6-Amidinium-2-(1-phenyl-1H-1,2,3-triazole-4-yl)benzothiazole methanesulfonate (13a)

According to the above-mentioned general method, 1-phenyl-1*H*-1,2,3-triazole-4-carbaldehyde **6a** (87 mg, 0.5 mmol) and 2-amino-5-amidiniumbenzenethiolate **7** (84 mg, 0.5 mmol) were used and refluxed for 2 h, giving 126 mg (0.394 mmol) of crude free base. Afterwards, the obtained free base was suspended in ethanol (10 ml) followed by the addition of methanesulfonic acid (29 µl, 0.45 mmol) and stirring at room temperature for 2 h. The reaction mixture was cooled overnight, the resulting precipitate was filtered off and dried at 75 °C. Yield of pure compound **13a** as colourless solid was 123 mg (76.8%), mp = 289–293 °C. ^1^H NMR (300 MHz, DMSO-d6) *δ* = 9.76 (s, 1H, Ar-*H*), 9.45 (s, 2H, -C(N*H*_2_)_2_^+^), 9.11 (s, 2H, -C(N*H*_2_)_2_^+^), 8.72 (s, 1H, Ar-*H*), 8.28 (d, 1H, *J* = 8.6 Hz, Ar-*H*), 8.06 (d, 2H, *J* = 7.6 Hz, Ar-*H*), 7.95 (d, 1H, *J* = 8.4 Hz, Ar-*H*), 7.70–7.56 (m, 3H, Ar-*H*), 2.35 (s, 3H, C*H*_3_SO_3_^–^). ^13^C NMR (75 MHz, DMSO-d6) *δ* = 165.4 (s), 162.7 (s), 156.3 (s), 142.4 (s), 136.1 (s), 134.2 (s), 130.0 (d, 2C), 129.5 (d), 126.5 (d), 125.3 (s), 123.6 (d), 122.8 (d), 122.7 (d), 120.6 (d, 2C), 39.8 (q). LC-MS (ESI) *m/z*: 321.2 (M + H^+^) calcd. for free base M = 320.08. Analysis calcd. for C_17_H_16_N_6_O_3_S_2_ (416.48): C, 49.23; H, 3.87; N, 20.18%; Found C, 49.28; H, 4.02; N, 20.01%.

#### 6-Amidinium-2-[1-(4-chlorophenyl)-1H-1,2,3-triazole-4-yl]benzothiazole methanesulfonate (13b)

According to the above-mentioned general method, 1-(4-chlorophenyl)-1*H*-1,2,3-triazole-4-carbaldehyde **6b** (104 mg, 0.5 mmol) and 2-amino-5-amidiniumbenzenethiolate **7** (84 mg, 0.5 mmol) were used and refluxed for 3 h, giving 151 mg (0.394 mmol) of crude free base. Afterwards, the obtained free base was suspended in ethanol (10 ml) followed by the addition of methanesulfonic acid (29 µl, 0.45 mmol) and stirring at room temperature for 2 h. The reaction mixture was cooled overnight, the resulting precipitate was filtered off and dried at 75 °C. Yield of pure compound **13b** as colourless solid was 143 mg (63.6%), mp > 300 °C. ^1^H NMR (300 MHz, DMSO-d6) *δ* = 9.70 (s, 1H, Ar-*H*), 9.18 (s, 4H, -C(N*H*_2_)_2_^+^), 8.70 (s, 1H, Ar-*H*), 8.25 (d, 1H, *J* = 8.3 Hz, Ar-*H*), 8.08 (d, 2H, *J* = 8.0 Hz, Ar-*H*), 7.95 (d, 1H, *J* = 7.8 Hz, Ar-*H*), 7.73 (d, 2H, *J* = 7.6 Hz, Ar-*H*), 2.34 (s, 3H, C*H*_3_SO_3_^–^). ^13^C NMR (75 MHz, DMSO-d6) *δ* = 165.1 (s), 162.2 (s), 155.9 (s), 142.2 (s), 134.7 (s), 134.0 (s), 133.6 (s), 129.5 (d, 2C), 126.0 (d), 125.2 (s), 123.0 (d), 122.5 (d), 122.1 (d, 2C), 39.4 (q). LC-MS (ESI) *m/z*: 355.1 (M + H^+^) calcd. for free base M = 354.05. Analysis calcd. for C_17_H_15_ClN_6_O_3_S_2_ (450.92): C, 45.28; H, 3.35; N, 18.64%; Found C, 45.28; H, 3.21; N, 18.71%.

#### 6-Amidinium-2-[1-(4-methoxyphenyl)-1H-1,2,3-triazole-4-yl]benzothiazole methanesulfonate (13c)

According to the above-mentioned general method, 1-(4-methoxyphenyl)-1*H*-1,2,3-triazole-4-carbaldehyde **6c** (102 mg, 0.5 mmol) and 2-amino-5-amidiniumbenzenethiolate **7** (84 mg, 0.5 mmol) were used and refluxed for 2 h, giving 130 mg (0.371 mmol) of crude free base. Afterwards, the obtained free base was suspended in ethanol (10 ml) followed by the addition of methanesulfonic acid (26 µl, 0.40 mmol) and stirring at room temperature for 2 h. The reaction mixture was cooled overnight, the resulting precipitate was filtered off and dried at 75 °C. Yield of pure compound **13c** as colourless solid was 114 mg (50.0%), mp > 300 °C. ^1^H NMR (300 MHz, DMSO-d6) *δ* = 9.65 (s, 1H, Ar-*H*), 9.45 (s, 2H, -C(N*H*_2_)_2_^+^), 9.11 (s, 2H, -C(N*H*_2_)_2_^+^), 8.71 (s, 1H, Ar-*H*), 8.27 (d, 1H, *J* = 8.1 Hz, Ar-*H*), 8.08–7.87 (m, 3H, Ar-*H*), 7.20 (d, 2H, *J* = 7.1 Hz, Ar-*H*), 3.86 (s, 3H, -OC*H*_3_), 2.35 (s, 3H, C*H*_3_SO_3_^–^). ^13^C NMR (151 MHz, DMSO-d6) *δ* = 165.3 (s), 162.8 (s), 159.8 (s), 156.2 (s), 142.1 (s), 134.1 (s), 129.4 (s), 126.3 (d), 125.1 (s), 123.4 (d), 122.7 (d), 122.5 (d), 122.2 (d, 2C), 114.9 (d, 2C), 55.6 (q), 39.7 (q). LC-MS (ESI) *m/z*: 351.1 (M + H^+^) calcd. for free base M = 350.09. Analysis calcd. for C_18_H_18_N_6_O_4_S_2_ × 0.5 H_2_O (455.51): C, 47.46; H, 4.20; N, 18.45%; Found C, 47.62; H, 4.12; N, 18.35%.

#### 6-Amidinium-2-(1-benzyl-1H-1,2,3-triazole-4-yl)benzothiazole methanesulfonate (13d)

According to the above-mentioned general method, 1-benzyl-1*H*-1,2,3-triazole-4-carbaldehyde **6d** (94 mg, 0.5 mmol) and 2-amino-5-amidiniumbenzenethiolate **7** (84 mg, 0.5 mmol) were used and refluxed for 3 h, giving 78 mg (0.233 mmol) of crude free base. Afterwards, the obtained free base was suspended in ethanol (5 ml) followed by the addition of methanesulfonic acid (16 µl, 0.25 mmol) and stirring at room temperature for 2 h. The reaction mixture was cooled overnight, the resulting precipitate was filtered off and dried at 75 °C. Yield of pure compound **13d** as colourless solid was 80 mg (35.7%), mp = 262–267 °C. ^1^H NMR (300 MHz, DMSO-d6) *δ* = 9.33 (s, 2H, -C(N*H*_2_)_2_^+^), 9.02 (s, 1H, Ar-*H*), 8.96 (s, 2H, -C(N*H*_2_)_2_^+^), 8.66 (s, 1H, Ar-*H*), 8.21 (d, 1H, *J* = 8.5 Hz, Ar-*H*), 7.94 (d, 1H, *J* = 8.6 Hz, Ar-*H*), 7.43–7.35 (m, 5H, Ar-*H*), 5.75 (s, 2H, -C*H*_2_-), 2.36 (s, 3H, C*H*_3_SO_3_^–^). ^13^C NMR (151 MHz, DMSO-d6) *δ* = 165.3 (s), 163.0 (s), 156.2 (s), 141.6 (s), 135.3 (s), 134.0 (s), 128.7 (d, 2C), 128.3 (d), 128.0 (d, 2C), 126.2 (d), 125.0 (s), 124.6 (d), 123.3 (d), 122.6 (d), 53.4 (t), 39.7 (q). LC-MS (ESI) *m/z*: 335.2 (M + H^+^) calcd. for free base M = 334.10. Analysis calcd. for C_18_H_18_N_6_O_3_S_2_ × H_2_O (448.52): C, 48.20; H, 4.49; N, 18.74%; Found C, 48.17; H, 4.38; N, 18.79%.

#### 6-(4,5-Dihydro-1H-imidazol-3-ium-2-yl)-2-(1-phenyl-1H-1,2,3-triazole-4-yl)benzothiazole methanesulfonate (14a)

According to the above-mentioned general method, 1-phenyl-1*H*-1,2,3-triazole-4-carbaldehyde **6a** (87 mg, 0.5 mmol) and 2-amino-5-(4,5-dihydro-1*H*-imidazol-3-ium-2-yl)benzenethiolate hydrate **8** (106 mg, 0.5 mmol) were used and refluxed for 3 h, giving 137 mg (0.396 mmol) of crude free base. Afterwards, the obtained free base was suspended in ethanol (10 ml) followed by the addition of methanesulfonic acid (29 µl, 0.44 mmol) and stirring at room temperature for 2 h. The reaction mixture was cooled overnight, the resulting precipitate was filtered off and dried at 75 °C. Yield of pure compound **14a** as colourless solid was 147 mg (66.5%), mp = 283–287 °C. ^1^H NMR (300 MHz, DMSO-d6) *δ* = 10.56 (s, 2H, C(N*H*-)_2_^+^), 9.70 (s, 1H, Ar-*H*), 8.81 (s, 1H, Ar-*H*), 8.31 (d, 1H, *J* = 8.6 Hz, Ar-*H*), 8.13–8.04 (m, 3H, Ar-*H*), 7.69–7.56 (m, 3H, Ar-*H*), 4.08 (s, 4H, -C*H*_2_C*H*_2_-), 2.33 (s, 3H, C*H*_3_SO_3_^–^). ^13^C NMR (151 MHz, DMSO-d6) *δ* = 164.7 (s), 162.9 (s), 156.4 (s), 141.9 (s), 135.8 (s), 134.3 (s), 129.5 (d, 2C), 129.0 (d), 126.1 (d), 123.4 (d), 122.8 (d), 122.4 (d), 120.3 (d, 2C), 118.8 (s), 44.3 (t, 2C). LC-MS (ESI) *m/z*: 347.2 (M + H^+^) calcd. for free base M = 346.10. Analysis calcd. for C_19_H_18_N_6_O_3_S_2_ (442.51): C, 51.57; H, 4.10; N, 18.99%; Found C, 51.67; H, 4.09; N, 18.87%.

#### 6-(4,5-Dihydro-1H-imidazol-3-ium-2-yl)-2-[1-(4-chlorophenyl)-1H-1,2,3-triazole-4-yl]benzothiazole methanesulfonate (14b)

According to the above-mentioned general method, 1-(4-chlorophenyl)-1*H*-1,2,3-triazole-4-carbaldehyde **6b** (104 mg, 0.5 mmol) and 2-amino-5-(4,5-dihydro-1*H*-imidazol-3-ium-2-yl)benzenethiolate hydrate **8** (106 mg, 0.5 mmol) were used and refluxed for 3 h, giving 138 mg (0.362 mmol) of crude free base. Afterwards, the obtained free base was suspended in ethanol (10 ml) followed by the addition of methanesulfonic acid (26 µl, 0.40 mmol) and stirring at room temperature for 2 h. The reaction mixture was cooled overnight, the resulting precipitate was filtered off and dried at 75 °C. Yield of pure compound **14b** as colourless solid was 144 mg (60.5%), mp > 300 °C. ^1^H NMR (300 MHz, DMSO-d6) *δ* = 10.55 (s, 2H, C(N*H*-)_2_^+^), 9.73 (s, 1H, Ar-*H*), 8.80 (s, 1H, Ar-*H*), 8.31 (d, 1H, *J* = 8.5 Hz, Ar-*H*), 8.11–8.05 (m, 3H, Ar-*H*), 7.74 (d, 2H, *J* = 8.5 Hz, Ar-*H*), 4.08 (s, 4H, -C*H*_2_C*H*_2_-), 2.32 (s, 3H, C*H*_3_SO_3_^–^). ^13^C NMR (75 MHz, DMSO-d6) *δ* = 164.7 (s), 162.9 (s), 156.4 (s), 142.1 (s), 134.7 (s), 134.3 (s), 133.6 (s), 129.6 (d, 2C), 126.3 (d), 123.6 (d), 123.0 (d), 122.7 (d), 122.1 (d, 2C), 118.9 (s), 44.4 (t, 2C). LC-MS (ESI) *m/z*: 381.2 (M + H^+^) calcd. for free base M = 380.06. Analysis calcd. for C_19_H_17_ClN_6_O_3_S_2_ (476.96): C, 47.85; H, 3.59; N, 17.62%; Found C, 47.83; H, 3.55; N, 17.75%.

#### 6-(4,5-Dihydro-1H-imidazol-3-ium-2-yl)-2-[1-(4-methoxyphenyl)-1H-1,2,3-triazole-4-yl]benzothiazole methanesulfonate (14c)

According to the above-mentioned general method, 1-(4-methoxyphenyl)-1*H*-1,2,3-triazole-4-carbaldehyde **6c** (102 mg, 0.5 mmol) and 2-amino-5-(4,5-dihydro-1*H*-imidazol-3-ium-2-yl)benzenethiolate hydrate **8** (106 mg, 0.5 mmol) were used and refluxed for 3 h, giving 132 mg (0.351 mmol) of crude free base. Afterwards, the obtained free base was suspended in ethanol (10 ml) followed by the addition of methanesulfonic acid (25 µl, 0.39 mmol) and stirring at room temperature for 2 h. The reaction mixture was cooled overnight, the resulting precipitate was filtered off and dried at 75 °C. Yield of pure compound **14c** as colourless solid was 110 mg (46.6%), mp > 300 °C. ^1^H NMR (300 MHz, DMSO-d6) *δ* = 10.61 (s, 2H, C(N*H*-)_2_^+^), 9.66 (s, 1H, Ar-*H*), 8.80 (s, 1H, Ar-*H*), 8.31 (d, 1H, *J* = 7.8 Hz, Ar-*H*), 8.05 (d, 1H, *J* = 7.3 Hz, Ar-*H*), 7.97 (d, 2H, *J* = 7.7 Hz, Ar-*H*), 7.20 (d, 2H, *J* = 7.5 Hz, Ar-*H*), 4.07 (s, 4H, -C*H*_2_C*H*_2_-), 3.86 (s, 3H, OC*H*_3_), 2.32 (s, 3H, C*H*_3_SO_3_^–^). ^13^C NMR (151 MHz, DMSO-d6) *δ* = 164.7 (s), 163.2 (s), 159.7 (s), 156.5 (s), 141.9 (s), 134.3 (s), 129.7 (s), 126.3 (d), 123.5 (d), 122.9 (d), 122.4 (d), 122.0 (d, 2C), 118.8 (s), 114.8 (d, 2C), 55.5 (q), 44.4 (t, 2C). LC-MS (ESI) *m/z*: 377.2 (M + H^+^) calcd. for free base M = 376.11. Analysis calcd. for C_20_H_20_N_6_O_4_S_2_ (472.54): C, 50.83; H, 4.27; N, 17.78%; Found C, 50.74; H, 4.33; N, 17.62%.

#### 6-(4,5-Dihydro-1H-imidazol-3-ium-2-yl)-2-(1-benzyl-1H-1,2,3-triazole-4-yl)benzothiazole methanesulfonate (14d)

According to the above-mentioned general method, 1-benzyl-1*H*-1,2,3-triazole-4-carbaldehyde **6d** (94 mg, 0.5 mmol) and 2-amino-5-(4,5-dihydro-1*H*-imidazol-3-ium-2-yl)benzenethiolate hydrate **8** (106 mg, 0.5 mmol) were used and refluxed for 3 h, giving 75 mg (0.233 mmol) of crude free base. Afterwards, the obtained free base was suspended in 2-propanol (5 ml) followed by the addition of methanesulfonic acid (16 µl, 0.25 mmol) and stirring at room temperature for 2 h. The reaction mixture was cooled overnight, the resulting precipitate was filtered off and dried at 75 °C. Yield of pure compound **14d** as colourless solid was 75 mg (32.9%), mp = 236–240 °C. ^1^H NMR (300 MHz, DMSO-d6) *δ* = 10.52 (s, 2H, C(N*H*-)_2_^+^), 9.09 (s, 1H, Ar-*H*), 8.76 (s, 1H, Ar-*H*), 8.27 (d, 1H, *J* = 8.3 Hz, Ar-*H*), 8.03 (d, 1H, *J* = 8.8 Hz, Ar-*H*), 7.47–7.35 (m, 5H, Ar-*H*), 5.75 (s, 2H, -C*H*_2_-), 4.07 (s, 4H, -C*H*_2_C*H*_2_-), 2.32 (s, 3H, C*H*_3_SO_3_^–^). ^13^C NMR (151 MHz, DMSO-d6) *δ* = 164.8 (s), 163.6 (s), 156.6 (s), 141.5 (s), 135.3 (s), 134.3 (s), 128.7 (d, 2C), 128.3 (d), 128.0 (d, 2C), 126.4 (d), 124.8 (d), 123.7 (d), 123.1 (d), 118.9 (s), 53.4 (t), 44.5 (t, 2C), 39.7 (q). LC-MS (ESI) *m/z*: 361.2 (M + H^+^) calcd. for free base M = 360.12. Analysis calcd. for C_20_H_20_N_6_O_3_S_2_ (456.54): C, 52.62; H, 4.42; N, 18.41%; Found C, 52.69; H, 4.36; N, 18.55%.

#### 6-(3,4,5,6-Tetrahydropyrimidin-1-ium-2-yl)-2-(1-phenyl-1H-1,2,3-triazole-4-yl)benzothiazole methanesulfonate (15a)

According to the above-mentioned general method, 1-phenyl-1*H*-1,2,3-triazole-4-carbaldehyde **6a** (87 mg, 0.5 mmol) and 2-amino-5-(3,4,5,6-tetrahydropyrimidin-1-ium-2-yl)benzenethiolate **9** (106 mg, 0.5 mmol) were used and refluxed for 3 h, giving 111 mg (0.308 mmol) of crude free base. Afterwards, the obtained free base was suspended in 2-propanol (10 ml) followed by the addition of methanesulfonic acid (22 µl, 0.34 mmol) and stirring at room temperature for 2 h. The reaction mixture was cooled overnight, the resulting precipitate was filtered off crystallised from ethanol/diethyl-ether mixture and dried at 75 °C. Yield of pure compound **15a** as colourless solid was 117 mg (47.6%), mp = 269–274 °C. ^1^H NMR (300 MHz, DMSO-d6) *δ* = 9.94 (s, 2H, C(N*H*-)_2_^+^), 9.62 (s, 1H, Ar-*H*), 8.60 (d, 1H, *J* = 1.5 Hz, Ar-*H*), 8.27 (d, 1H, *J* = 8.5 Hz, Ar-*H*), 8.04 (d, 2H, *J* = 7.9 Hz, Ar-*H*), 7.88 (dd, 1H, *J* = 1.9 Hz, *J* = 8.6 Hz, Ar-*H*), 7.69–7.56 (m, 3H, Ar-*H*), 3.57 (t, 4H, *J* = 5.7 Hz, -C*H*_2_CH_2_C*H*_2_-), 2.32 (s, 3H, C*H*_3_SO_3_^–^), 2.06 (m, 2H, -CH_2_C*H*_2_CH_2_-). ^13^C NMR (151 MHz, DMSO-d6) *δ* = 162.3 (s), 159.1 (s), 155.8 (s), 142.3 (s), 136.0 (s), 134.1 (s), 129.8 (d, 2C), 129.3 (d), 125.9 (d), 125.5 (s), 122.8 (d), 122.7 (d), 122.5 (d), 120.5 (d, 2C), 39.6 (q), 38.8 (t, 2C), 17.6 (t). LC-MS (ESI) *m/z*: 361.2 (M + H^+^) calcd. for free base M = 360.12. Analysis calcd. for C_20_H_20_N_6_O_3_S_2_ × 2H_2_O (492.57): C, 48.77; H, 4.91; N, 17.06%; Found C, 48.63; H, 5.02; N, 17.04%.

#### 6-(3,4,5,6-Tetrahydropyrimidin-1-ium-2-yl)-2-[1-(4-chlorophenyl)-1H-1,2,3-triazole-4-yl]benzothiazole methanesulfonate (15b)

According to the above-mentioned general method, 1-(4-chlorophenyl)-1*H*-1,2,3-triazole-4-carbaldehyde **6b** (104 mg, 0.5 mmol) and 2-amino-5-(3,4,5,6-tetrahydropyrimidin-1-ium-2-yl)benzenethiolate **9** (106 mg, 0.5 mmol) were used and refluxed for 2 h, giving 180 mg (0.457 mmol) of crude free base. Afterwards, the obtained free base was suspended in ethanol (10 ml) followed by the addition of methanesulfonic acid (34 µl, 0.52 mmol) and stirring at room temperature for 2 h. The reaction mixture was cooled overnight, the resulting precipitate was filtered off and dried at 75 °C. Yield of pure compound **15b** as colourless solid was 143 mg (58.4%), mp = 288–293 °C. ^1^H NMR (300 MHz, DMSO-d6) *δ* = 9.99 (s, 2H, C(N*H*-)_2_^+^), 9.71 (s, 1H, Ar-*H*), 8.61 (s, 1H, Ar-*H*), 8.27 (d, 1H, *J* = 8.5 Hz, Ar-*H*), 8.10 (d, 2H, *J* = 8.7 Hz, Ar-*H*), 7.87 (d, 1H, *J* = 7.6 Hz, Ar-*H*), 7.74 (d, 2H, *J* = 8.7 Hz, Ar-*H*), 3.56 (t, 4H, *J* = 5.4 Hz, -C*H*_2_CH_2_C*H*_2_-), 2.31 (s, 3H, C*H*_3_SO_3_^–^), 2.04 (m, 2H, -CH_2_C*H*_2_CH_2_-). ^13^C NMR (151 MHz, DMSO-d6) *δ* = 162.0 (s), 159.0 (s), 155.7 (s), 142.3 (s), 134.8 (s), 134.1 (s), 133.6 (s), 129.7 (d, 2C), 125.8 (d), 125.6 (s), 122.7 (d), 122.6 (d), 122.2 (d, 2C), 39.5 (q), 38.8 (t, 2C), 17.6 (t). LC-MS (ESI) *m/z*: 395.2 (M + H^+^) calcd. for free base M = 394.08. Analysis calcd. for C_20_H_19_ClN_6_O_3_S_2_ (490.99): C, 48.92; H, 3.90; N, 17.12%; Found C, 49.03; H, 3.66; N, 17.12%.

#### 6-(3,4,5,6-Tetrahydropyrimidin-1-ium-2-yl)-2-[1-(4-methoxyphenyl)-1H-1,2,3-triazole-4-yl]benzothiazole methanesulfonate (15c)

According to the above-mentioned general method, 1-(4-methoxyphenyl)-1*H*-1,2,3-triazole-4-carbaldehyde **6c** (102 mg, 0.5 mmol) and 2-amino-5-(3,4,5,6-tetrahydropyrimidin-1-ium-2-yl)benzenethiolate **9** (106 mg, 0.5 mmol) were used and refluxed for 2 h, giving 145 mg (0.371 mmol) of crude free base. Afterwards, the obtained free base was suspended in ethanol (10 ml) followed by the addition of methanesulfonic acid (26 µl, 0.40 mmol) and stirring at room temperature for 2 h. The reaction mixture was cooled overnight, the resulting precipitate was filtered off and dried at 75 °C. Yield of pure compound **15c** as colourless solid was 141 mg (55.3%), mp = 261–265 °C. ^1^H NMR (300 MHz, DMSO-d6) *δ* = 9.99 (s, 2H, C(N*H*-)_2_^+^), 9.57 (s, 1H, Ar-*H*), 8.60 (s, 1H, Ar-*H*), 8.26 (d, 1H, *J* = 8.5 Hz, Ar-*H*), 7.95 (d, 2H, *J* = 8.7 Hz, Ar-*H*), 7.86 (d, 1H, *J* = 8.3 Hz, Ar-*H*), 7.20 (d, 2H, *J* = 8.6 Hz, Ar-*H*), 3.87 (s, 3H, OC*H*_3_), 3.56 (m, 4H, -C*H*_2_CH_2_C*H*_2_-), 2.30 (s, 3H, C*H*_3_SO_3_^–^), 2.04 (m, 2H, -CH_2_C*H*_2_CH_2_-). ^13^C NMR (75 MHz, DMSO-d6) *δ* = 162.3, 159.7, 159.1, 155.7, 142.0, 134.1, 129.3, 125.7, 125.4, 122.7, 122.5, 122.3, 122.1 (2C), 114.8 (2C), 55.5, 38.8, 17.5. LC-MS (ESI) *m/z*: 391.2 (M + H^+^) calcd. for free base M = 390.13. Analysis calcd. for C_21_H_22_N_6_O_4_S_2_ × 1.25 H_2_O: C, 49.54; H, 4.85; N, 16.51%; Found C, 49.41; H, 4.83; N, 17.63%.

#### 6-(3,4,5,6-Tetrahydropyrimidin-1-ium-2-yl)-2-(1-benzyl-1H-1,2,3-triazole-4-yl)benzothiazole methanesulfonate (15d)

According to the above-mentioned general method, 1-benzyl-1*H*-1,2,3-triazole-4-carbaldehyde **6d** (94 mg, 0.5 mmol) and 2-amino-5-(3,4,5,6-tetrahydropyrimidin-1-ium-2-yl)benzenethiolate **9** (106 mg, 0.5 mmol) were used and refluxed for 2 h, giving 106 mg (0.283 mmol) of crude free base. Afterwards, the obtained free base was suspended in 2-propanol (5 ml) followed by the addition of methanesulfonic acid (14 µl, 0.21 mmol) and stirring at room temperature for 2 h. The reaction mixture was cooled overnight, the resulting precipitate was filtered off and dried at 75 °C. Yield of pure compound **14d** as colourless solid was 74 mg (32.9%), mp = 170–174 °C. ^1^H NMR (300 MHz, DMSO-d6) *δ* = 9.94 (s, 2H, C(N*H*-)_2_^+^), 9.01 (s, 1H, Ar-*H*), 8.56 (s, 1H, Ar-*H*), 8.21 (d, 1H, *J* = 8.4 Hz, Ar-*H*), 7.85 (d, 1H, *J* = 7.9 Hz, Ar-*H*), 7.47–7.34 (m, 5H, Ar-*H*), 5.75 (s, 2H, -C*H*_2_-), 3.56 (m, 4H, -C*H*_2_CH_2_C*H*_2_-), 2.32 (s, 3H, C*H*_3_SO_3_^–^), 2.05 (m, 2H, -CH_2_C*H*_2_CH_2_-). ^13^C NMR (151 MHz, DMSO-d6) *δ* = 162.7 (s), 159.1 (s), 155.8 (s), 141.6 (s), 135.3 (s), 134.0 (s), 128.7 (d, 2C), 128.3 (d), 128.0 (d, 2C), 125.8 (d), 125.5 (s), 124.6 (d), 122.8 (d), 122.7 (d), 53.4 (t), 38.9 (t, 2C), 17.6 (t). LC-MS (ESI) *m/z*: 375.2 (M + H^+^) calcd. for free base M = 374.13. Analysis calcd. for C_21_H_22_N_6_O_4_S_2_ × 1.5 H_2_O (497.59): C, 50.69; H, 5.06; N, 16.89%; Found C, 50.91; H, 5.14; N, 16.86%.

### Biological evaluations

#### Cell culturing

Human cell lines SW620 (colorectal adenocarcinoma, metastatic), MCF-7 (human breast adenocarcinoma), CFPAC-1 (pancreatic adenocarcinoma), HeLa (cervical carcinoma), and HFF-1 (human foreskin fibroblasts) were obtained from the American Type Culture Collection (ATCC). Cells were cultured in a humidified atmosphere at 37 °C with 5% CO_2_. As a growth medium, Dulbecco’s modified Eagle medium (DMEM) was used with the addition of foetal bovine serum (10%), L-glutamine (2 mM), and antibiotics: streptomycin (100 mg/ml) and penicillin (100 U/ml).

#### Proliferation assay

Cells were seeded onto 96-well microtiter plates at a seeding density of 3000 cells/well for carcinoma cell lines, and 5000 cells/well for normal human fibroblasts. The next day, cells were treated with test agents at five different concentrations (0.01–100 µM) and further incubated for 72 h. DMSO (solvent) was tested for potential cytotoxic effect but it did not exceed 0.1%. 5-Fluorouracil (5-FU, 0.384 M fluorouracilum, Pliva 500 mg/10 ml) dissolved in physiological solution was used as a positive control. Following 72 h incubation, the MTT assay was performed and measured absorbances were transformed into a percentage of cell growth as described previously^47^. Results were obtained from three independent experiments. IC_50_ values were calculated using linear regression analysis (FORECAST option taking into account the concentration range of two experimental points above and below IC_50_).

### Antitrypanosomal screening and cytotoxicity assays

#### Antitrypanosomal screening

Bloodstream form *T. b. brucei* (strain 221) were cultured in modified Iscove’s medium^48^ and assays were carried out in 96-well microtiter plates (200 μl volumes) to determine the IC_50_ and IC_90_ values of each compound. Parasites growth was initiated at 2.5 × 10^4^ ml^−1^, compounds were added at a range of concentrations, and the plates were incubated at 37 °C. Resazurin (20 μl at 0.125 mg ml^−1^) was added after 48 h, the plates were incubated for a further 16 h, and then read in a Spectramax plate reader, and data analysed using GraphPad Prism. Each drug was tested in triplicate.

#### L6 cell proliferation

For cytotoxicity assays, L6 cells (a rat myoblast line) were seeded into 96-well microtiter plates at 1 × 10^4^ ml^−1^ in 200 μl of the growth medium, and different compound concentrations were added. The plates were then incubated for 6 days at 37 °C and 20 μl resazurin was added to each well. After a further 8 h incubation, the fluorescence was determined using a Spectramax plate reader, as outlined above.

### DNA binding study

Compounds **11a**, **11b**, **12b**, **14a**, **14c** and **15b** were dissolved (*c* = 5 × 10^−3 ^M for **12b**, **14a**, **14c**, **15b**, *c* = 4 × 10^−3 ^M for **11a**, **11b**) in water, while compounds **10b** and **14b** were dissolved (*c* = 5 × 10^−3 ^M) in DMSO. These solutions were used for measurements in an aqueous buffer (pH = 7, sodium cacodylate buffer, *I* = 0.05 mol dm^−3^). Polynucleotides were purchased as noted: poly (dAdT)_2_ and calf thymus ctDNA (Sigma-Aldrich). Polynucleotides were dissolved in Na-cacodylate buffer, *I* = 0.05 mol dm^−3^, pH = 7. The calf thymus ctDNA was additionally sonicated and filtered through a 0.45 mm filter^49^. Polynucleotide concentration was determined spectroscopically^50^^,^^51^ as the concentration of phosphates.

#### UV/vis measurements

The UV/Vis spectra were recorded on a Varian Cary 100 Bio spectrophotometer using 1 cm path quartz cuvettes. Calibration experiments were performed at 25 °C and pH = 7 (*I* = 0.05 mol dm^−3^, sodium cacodylate buffer). Absorption maxima and corresponding molar extinction coefficients (ε) of benzothiazole derivatives are given in Table S1 (Supplementary material). Thermal melting curves for DNA and their complexes with studied compounds were determined as previously described by following the absorption change at 260 nm as a function of temperature. The absorbance of the ligands was subtracted from every curve and the absorbance scale was normalised. *T*_m_ values are the midpoints of the transition curves determined from the maximum of the first derivative and checked graphically by the tangent method. The Δ*T*_m_ values were calculated by subtracting *T*_m_ of the free nucleic acid from *T*_m_ of the complex. Every Δ*T*_m_ value reported here was the average of at least two measurements. The error in Δ*T*_m_ is ±0.5 °C.

#### Fluorimetric measurements

Fluorescence spectra were recorded on a Varian Cary Eclipse spectrophotometer at 25 °C using appropriate 1 cm path quartz cuvettes. Fluorimetric experiments were performed at pH = 7 (*I* = 0.05 mol dm^−3^, sodium cacodylate buffer) by adding portions of polynucleotide solution into the solution of the studied compound. In fluorimetric experiments, an excitation wavelength of *λ*_exc_ ≥300 nm was used to avoid the inner filter effect caused due to increasing absorbance of the polynucleotide. Emissions were determined in the range *λ*_em_ = 350–650 nm. Values for *K*_s_ were obtained by processing titration data using the Scatchard equation^52^. All had satisfactory correlation coefficients (>0.99).

#### CD measurements

CD spectra were recorded on a JASCO J815 spectrophotometer in 1 cm path quartz cuvettes. CD parameters: range = 500–220 nm, data pitch = 2, standard sensitivity, scanning speed = 200 nm/min, accumulation = 3–5. Titrations were performed at 25 °C and pH = 7 (*I* = 0.05 mol dm^−3^, sodium cacodylate buffer). CD experiments were done by adding portions of the compound stock solution into the polynucleotide solution.

## Results and discussion

### Chemistry

**Scheme 1.**
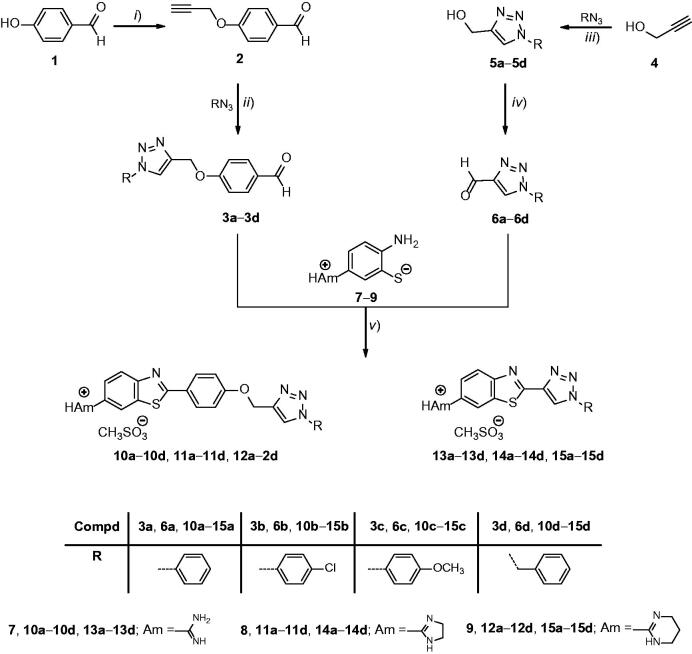
Reagents and reaction conditions: (*i*): propargyl bromide, K_2_CO_3,_ EtOH, reflux; (*ii*): corresponding azides, CuSO_4_, Cu(0), DMF, t-BuOH:H_2_O = 1:1, 80 °C; (*iii*): corresponding azides, Cu(OAc)_2_, MeOH, 80 °C, US, 1.5 h; (*iv*): (COCl)_2_, DMSO, Et_3_N, CH_2_Cl_2_, −78 °C to rt 45 min; (*v*): (a) corresponding thiolates **7–9** and aldehydes **3a–3d**, **6a–6d**, HOAc, reflux 2–4 h then NaOH (aq) pH 10–12, (b) corresponding amidine free bases, EtOH or *i*-PrOH, 1.1 eq. MSA, 2 h, rt.

The synthesis of novel 1,2,3-triazolyl linked 6-amidino substituted benzothiazole derivatives **10a–10d**, **11a–11d**, **12a–12d**, **13a–13d**, **14a–14d**, **15a–15d** were synthesised according to the procedure shown in Scheme 1.

4-(1,2,3-Triazol-1-yl)benzaldehyde derivatives (**3a–3d**) were prepared by propargylation of 4-hydroxy benzaldehyde to give 4-*O*-propargylated benzaldehyde (**2**). Intermediate **2** was then used as a dipolarophile in copper(I)-catalysed Huisgen 1,3-dipolar cycloaddition with unsubstituted, *p*-chloro- and *p*-methoxy-substituted phenyl azides to afford **3a–3d**. With the aim of assessing the influence of the phenoxymethylene linker between the benzothiazole and 1,2,3-triazole moieties on the anti-proliferative and antitrypanosomal activities, the 1,2,3-triazole ring was introduced directly (**13a–13d**, **14a–14d**, and **15a–15d**) to the benzothiazole ring as displayed in Scheme 1. 1-Aryl-substituted 1,2,3-triazolyl aliphatic alcohols (**5a–5d**) were prepared in excellent yields by using the ultrasound-assisted reaction of propargyl alcohol and corresponding aromatic azides with Cu(OAc)_2_. 1-Aryl-substituted 1,2,3-triazole-carbaldehyde precursors (**6a–6d**) were subsequently obtained by the Swern oxidation^53^.

Amidino-substituted 2-aminothiophenoles (**7–9**) were prepared from 6-cyanobenzothiazole by the Pinner method, as previously reported^45^^,^^46^. To study the influence of the type of the hydrophilic amidino substituents, non-substituted amidino, imidazolino and pyrimidino moieties were introduced at C-6 of benzothiazole scaffold. Condensation of various amidino-substituted 2-aminothiophenoles (**7–9**) with 4-(1,2,3-triazol-1-yl)benzaldehyde precursors (**3a–3d**) and 1,2,3-triazole-4-carbaldehyde precursors (**6a–6d**) was carried out in acetic acid, followed by a simple acid-base reaction step to afford the targeted amidino-substituted benzothiazole mesylates (**10a–10d**, **11a–11d**, **12a–12d**, **13a–13d**, **14a–14d**, **15a–15d)**.

The structures of novel 6-amidino-substituted benzothiazoles mesylates **10a–10d**, **11a–11d**, **12a–12d**, **13a–13d**, **14a–14d**, **15a–15d** were determined by using ^1^H and ^13^C NMR spectroscopy, mass spectrometry, and elemental analysis (Materials and methods section). The chemical shifts in the ^1^H and ^13^C NMR spectra and the H–H coupling constants were consistent with the proposed structures (Supplementary material).

### Biological evaluations

#### Evaluation of antiproliferative activity

Antiproliferative evaluations of all synthesised compounds were performed *in vitro* on human tumour cell lines, SW620 (colorectal adenocarcinoma, metastatic), MCF-7 (human breast adenocarcinoma), CFPAC-1 (pancreatic adenocarcinoma), HeLa (cervical carcinoma), and non-tumour HFF-1 (human foreskin fibroblasts) cells. 5-Fluorouracil (5-FU) was used as a reference drug. The results are presented in Table 1.

**Table 1.** The growth-inhibition effects^a^ *in vitro* of compounds **10a–10d**, **11a–11d**, **12a–12d**, **13a–13d**, **14a–14d**, **15a–15d** on human tumour cell lines and normal fibroblasts.
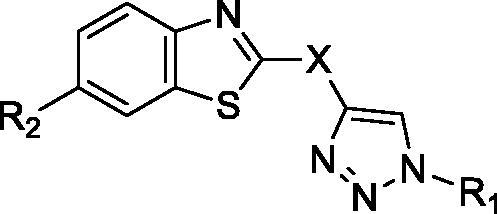

Compd.	R_1_	X	R_2_	IC_50_ (μM)				
				SW620	CFPAC-1	MCF-7	HeLa	HFF-1/WI-38^c^
**10a**	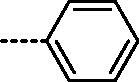	–PhOCH_2_–	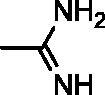	2.89 ± 0.03	3.34 ± 0.19	3.39 ± 1.17	2.89 ± 0.11	0.17 ± 0.04
**10b**	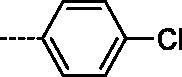	–PhOCH_2_–	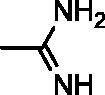	3.05 ± 0.82	1.76±.0.17	0.87 ± 0.18	1.24 ± 0.60	0.17 ± 0.13
**10c**	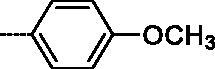	–PhOCH_2_–	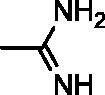	3.86 ± 0.29	3.45 ± 0.29	3.66 ± 1.49	2.87 ± 1.24	0.31 ± 0.08
**10d**	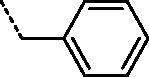	–PhOCH_2_–	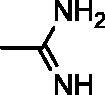	1.18 ± 0.18	5.15 ± 0.23	3.02 ± 0.98	1.77 ± 0.45	0.76 ± 0.18
**11a**	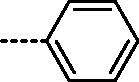	–PhOCH_2_–	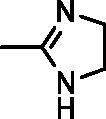	0.46 ± 0.03	0.45 ± 0.01	0.49 ± 0.01	0.57 ± 0.19	0.26 ± 0.02
**11b**	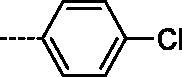	–PhOCH_2_–	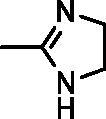	2.84 ± 0.19	3.09 ± 0.19	3.12 ± 0.36	2.61 ± 0.42	0.17 ± 0.09
**11c**	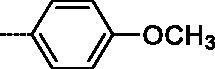	–PhOCH_2_–	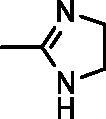	0.50 ± 0.16	2.06 ± 0.70	0.86 ± 0.97	2.53 ± 0.01	0.24 ± 0.13
**11d**	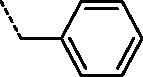	–PhOCH_2_–	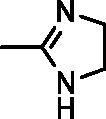	1.81 ± 0.55	1.21 ± 0.74	2.21 ± 1.17	1.40 ± 0.75	0.18 ± 0.02
**12a**	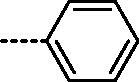	–PhOCH_2_–	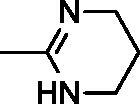	4.17 ± 0.28	3.98 ± 0.29	3.98 ± 0.04	2.95 ± 0.38	0.44 ± 0.03
**12b**	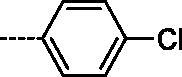	–PhOCH_2_–	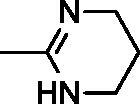	0.66 ± 0.21	3.55 ± 0.81	3.91 ± 1.19	2.81 ± 0.58	0.24 ± 0.09
**12c**	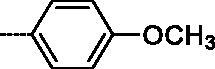	–PhOCH_2_–	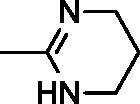	5.22 ± 1.24	3.31 ± 0.60	3.46 ± 0.45	2.64 ± 0.46	0.46 ± 0.27
**12d**	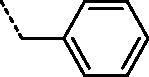	–PhOCH_2_–	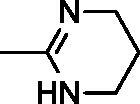	7.94 ± 2.58	23.42 ± 0.29	6.01 ± 0.53	8.60 ± 0.85	1.74 ± 0.29
**13a**	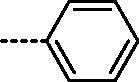		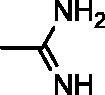	24.22 ± 4.31	26.58 ± 4.69	25.58 ± 3.15	7.77 ± 0.05	2.07 ± 0.13
**13b**	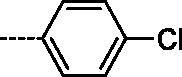		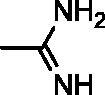	3.55 ± 0.85	3.17 ± 0.11	3.32 ± 0.01	2.74 ± 0.21	0.85 ± 0.05
**13c**	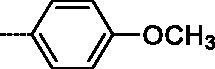		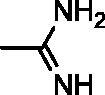	5.39 ± 0.41	5.23 ± 0.87	4.50 ± 0.43	3.64 ± 1.44	2.99 ± 1.17
**13d**	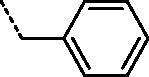		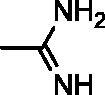	9.48 ± 0.29	25.93 ± 3.71	22.56 ± 0.91	14.90 ± 1.54	4.04 ± 1.35
**14a**	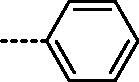		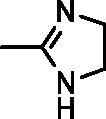	0.25 ± 0.16	0.45 ± 0.19	0.52 ± 0.26	0.67 ± 0.06	0.12 ± 0.01
**14b**	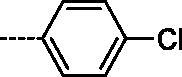		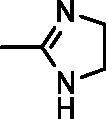	0.35 ± 0.13	2.42 ± 0.06	1.77 ± 0.18	0.48 ± 0.01	0.36 ± 0.18
**14c**	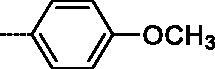		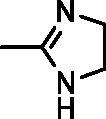	0.36 ± 0.09	0.48 ± 0.03	0.54 ± 0.01	0.38 ± 0.10	0.18 ± 0.01
**14d**	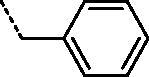		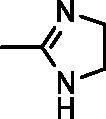	0.85 ± 0.61	8.71 ± 3.74	1.76 ± 1.17	0.82 ± 0.01	0.11 ± 0.01
**15a**	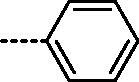		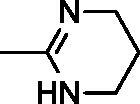	4.15 ± 1.58	37.57 ± 4.58	8.31 ± 0.12	7.62 ± 0.84	0.53 ± 0.10
**15b**	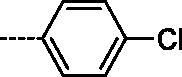		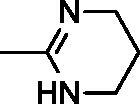	0.89 ± 0.31	0.71 ± 0.17	2.10 ± 1.09	2.27 ± 0.08	0.74 ± 0.05
**15c**	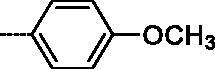		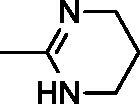	4.07 ± 1.06	4.74 ± 0.14	4.28 ± 0.06	4.30 ± 0.41	1.33 ± 0.17
**15d**	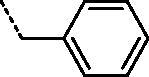		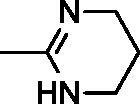	31.86 ± 8.38	49.89 ± 12.5	40.58 ± 10.6	30.99 ± 4.50	18.93 ± 0.19
**5-FU^b^**				6.4 ± 0.22	6.45 ± 0.66	0.09 ± 0.01	47.52 ± 1.93	0.94^c^

^a^50% inhibitory concentration or compound concentration required to inhibit tumour cell proliferation by 50%; ^b^control substance 5-fluorouracil; ^c^WI-38 fibroblast cell line.

**Figure 2.**
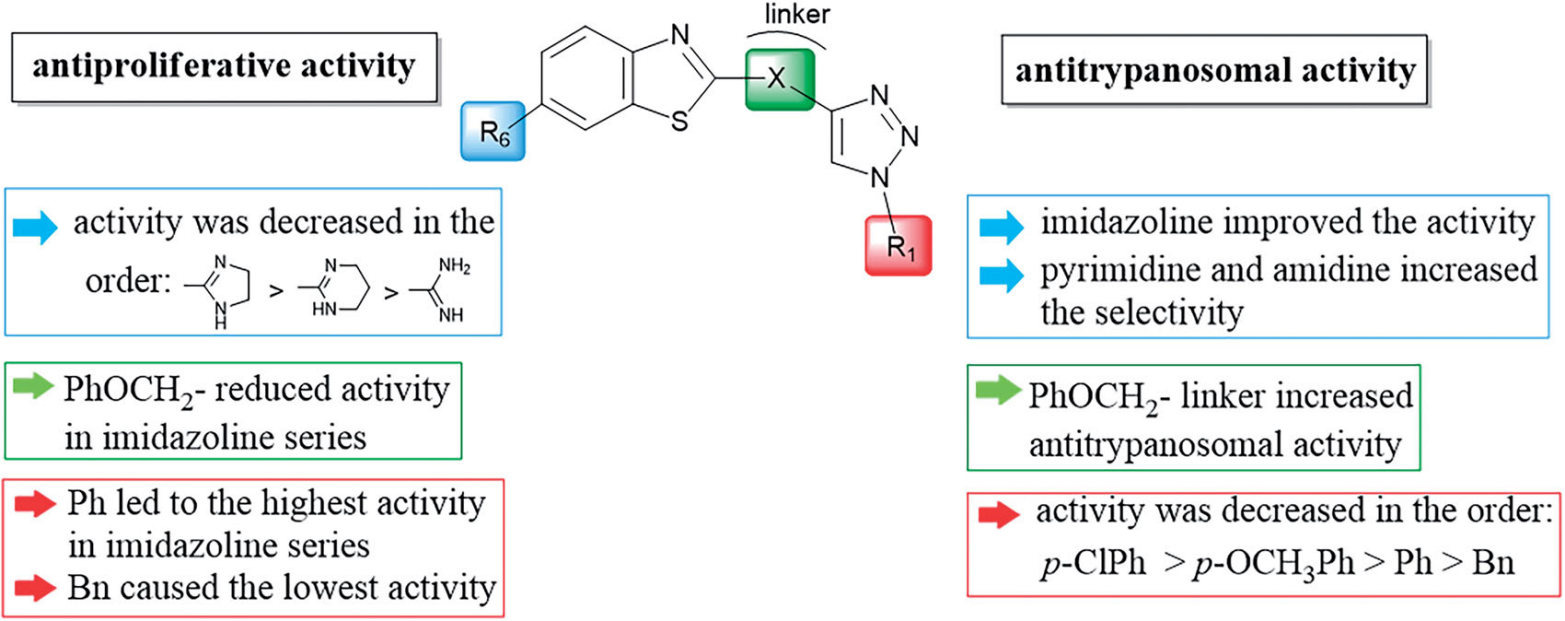
Structure–activity relationship for antiproliferative and antitrypanosomal activity of the 2-arylbenzothiazole amidines.

It can be observed that the tested compounds exhibited strong to moderate inhibitory activity towards tumour cell lines. However, compounds with pronounced antiproliferative effects affected the proliferation of the non-tumour HFF-1 cell line. The type of amidino moiety at the benzothiazole influenced the antiproliferative activities (Figure 2). For example, among the 6-amidino benzothiazoles, imidazolino-substituted derivatives **11a–11d** and **14a–14d** showed the best inhibitory effects, particularly on colorectal adenocarcinoma (SW620) and cervical carcinoma (HeLa) cells. This correlates with our earlier findings for the benzimidazole series^41^. Unsubstituted amidino-benzothiazole **13a–13d** exhibited up to 10-fold lower activities relative to their imidazolines congeners **14a–14d**. The cytostatic effects decreased in the following order: imidazoline > pyrimidine > non-substituted amidine.

The effect of the aromatic substituent at N-1 of 1,2,3-triazole ring on antiproliferative activities varied depending on the type of 6-amidino moiety. In most cases, the *p*-chlorophenyl aromatic unit contributed to the enhanced antiproliferative effects of **10b**, **12b**, **13b**, and **15b**, except in the case of imidazolino-benzothiazoles. In benzothiazole imidazolines, the unsubstituted phenyl ring in **11a** and **14a** had the highest influence on inhibitory activity. The 2-imidazolino-substituted derivative **14a** exhibited the highest activity on SW620 (IC_50_ = 0.25 µM) and CFPAC-1 (IC_50_ = 0.45 µM) cell lines, while **14c**, which contains a *p*-methoxyphenyl ring, was the most potent against HeLa cells (IC_50_ = 0.38 µM). The benzothiazole imidazoline **11a** was the most active representative of this class (IC_50_ = 0.49 µM) against MCF-7 cells. Comparison of the effect of the aryl substituents at the N-1 position of the 1,2,3-triazole ring on activity revealed that, among 2-imidazolino-substituted derivatives, phenyl and *p*-methoxyphenyl substituents caused better potencies than *p*-chlorophenyl and benzyl substituents.

The introduction of a phenoxymethylene linker improved the activity of the resulting **10a–10d** and **12a–12d** up to 8-fold, relative to the corresponding benzothiazoles directly connected to 1,2,3-triazole in **13a–13d** and **15a–15d**. In contrast, with imidazolino-benzothiazoles, direct fusion of benzothiazole to 1,2,3-triazole in **14a–14d** enhanced antiproliferative activity compared to **11a–11d,** which contained a phenoxymethylene spacer.

#### Evaluation of antitrypanosomal activity

Preliminary screening of 2-arylbenzimidazole amidines **6a**–**6c**, **7a**–**7c**, **8**, **14a**–**18a**, **14b**–**18b**, and **14c**–**18c** against bloodstream-form *T. brucei in vitro* was performed at a range of concentrations to select compounds with the highest inhibition for further evaluation (Table S1, Supplementary material). Thus, compounds, **10a–10c**, **11a–11d**, **12b**, **12c**, **13a**, **13b**, **14a–14d**, and **15b**, were submitted for more detailed evaluation and the concentrations that inhibited growth by 50% (IC_50_) and 90% (IC_90_) were determined (Table 2). Nifurtimox was included as a reference drug. Cytotoxicity was assessed using the rat myoblast cell line L6.

**Table 2.** Antitrypanosomal activity^a^ of compounds **10a–10c**, **11a–11d**, **12b–12c**, **13a–13b**, **14a–14d** and **15b** against *Trypanosoma brucei* (strain 221).
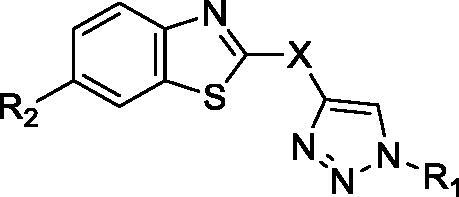

Compd.	R_1_	X	R_2_	*T. brucei*		L6 cells	SI^c^
				IC_50_ (µM)	IC_90_ (µM)	IC_50_ (µM)	IC_50_ (L6/Tb)
**10a**	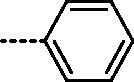	–PhOCH_2_–	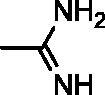	1.17 ± 0.10	1.45 ± 0.11	<2.0	
**10b**	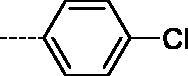	–PhOCH_2_–	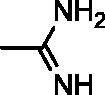	0.54 ± 0.13	0.81 ± 0.04	1.46 ± 0.45	2.7
**10c**	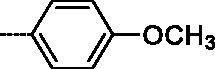	–PhOCH_2_–	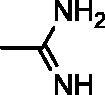	1.00 ± 0.07	1.26 ± 0.02	<2.0	
**11a**	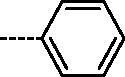	–PhOCH_2_–	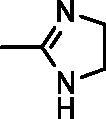	0.23 ± 0.02	0.28 ± 0.03	<0.2	
**11b**	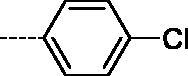	–PhOCH_2_–	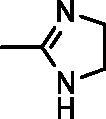	0.09 ± 0.02	0.12 ± 0.03	<2.0	
**11c**	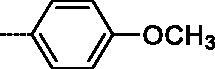	–PhOCH_2_–	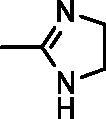	0.19 ± 0.04	0.23 ± 0.05	<2.0	
**11d**	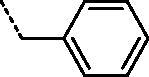	–PhOCH_2_–	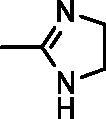	1.09 ± 0.02	1.30 ± 0.02	<0.2	
**12b**	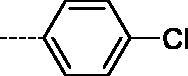	–PhOCH_2_–	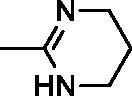	0.91 ± 0.08	1.19 ± 0.02	2.32 ± 0.18	2.5
**12c**	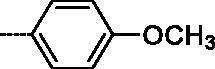	–PhOCH_2_–	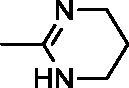	0.90 ± 0.05	1.18 ± 0.02	2.92 ± 0.26	3.2
**13a**	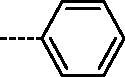		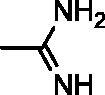	2.02 ± 0.10	2.52 ± 0.34	7.97 ± 0.96	3.9
**13b**	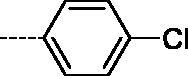		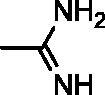	0.67 ± 0.07	1.02 ± 0.02	2.69 ± 0.42	4.0
**14a**	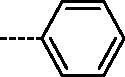		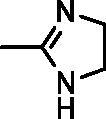	0.40 ± 0.02	0.53 ± 0.03	<2.0	
**14b**	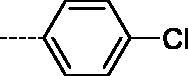		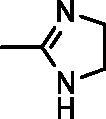	0.32 ± 0.06	0.48 ± 0.02	<2.0	
**14c**	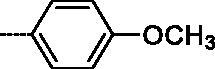		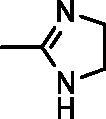	0.31 ± 0.02	0.49 ± 0.01	<2.0	
**14d**	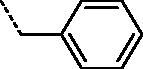		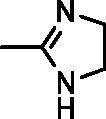	3.68 ± 0.22	8.66 ± 0.20	6.64 ± 0.53	1.8
**15b**	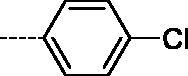		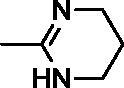	1.12 ± 0.23	3.84 ± 0.65	7.69 ± 0.16	6.9
**Nifurtimox^b^**				2.0 ± 0.24^b^			

^a^*In vitro* activity against bloodstream form *T. brucei* expressed as the concentration that inhibited growth by 50% (IC_50_) and 90% (IC_90_). Data are the mean of triplicate experiments ± SEM. ^b^Taken from Wilkinson et al.^54^. ^c^Selectivity index, SI = [IC_50_ L6 cells]/[IC_50_ *T. brucei*].

All tested compounds showed potent activity against *T. brucei* with IC_50_ values ranging from 0.09 to 3.68 µM and IC_90_s ranging from 0.12 to 8.66 µM, more potent than the front-line drug nifurtimox. We investigated the influence of unsubstituted and cyclic amidino moiety, and aromatic substituent attached to the N-1 of the 1,2,3-triazole ring on antitrypanosomal activity (Figure 2). Similar to antiproliferative evaluations, imidazolino-substituted benzothiazoles **11a–11c** and **14a–14c** exhibited the best anti-trypanosomal activity, with the *p*-chlorophenyl analogue **11b** being the most promising compound (IC_50_ = 0.09 µM, IC_90_ = 0.12 µM). With the exception of compounds **11d** and **14d**, which contain the N-1-benzyl-1,2,3-triazolyl moiety, benzothiazole imidazolines **11a–11c** and **14a–14c** showed potency in submicromolar concentrations, with IC_90_ in the range of 0.12–0.53 µM. However, these compounds were cytotoxic to rat myoblast cell line L6 (IC_50_ > 0.2 µM). Some selectivity (SI = 1.8–6.9) was observed for benzothiazole pyrimidines and non-substituted amidines.

Replacement of the phenoxymethylene linker in **10a–10d**, **11a–11d**, and **12a–12d** with a direct fusion of benzothiazole to 1,2,3-triazole in **13a–13d**, **14a–14d**, and **15a–15d** reduced antitrypanosomal activity against bloodstream-form *T. brucei*. Furthermore, the introduction of an electron-withdrawing *p*-chloro substituent in **10b–15b** improved the inhibitory effect. The relationship between the type of aromatic substituents and their activities revealed that antitrypanosomal effects decreased in the following order: *p*-ClPh>*p*-OCH_3_Ph > Ph > Bn.

#### DNA binding study

As a carrier of genetic information and a great influence on vital processes in the cell, DNA is the main target of a large number of drugs with anticancer and antiprotozoal activity^55–57^. Among them, anthracyclines that target DNA topoisomerase II cause highly lethal DNA breaks in proliferating cancer cells, while some drugs, such as quinacrine, with a long history of clinical use in the treatment of malaria, modulate cellular pathways that lead to cellular toxicity and death^55^.

**Figure 3.**
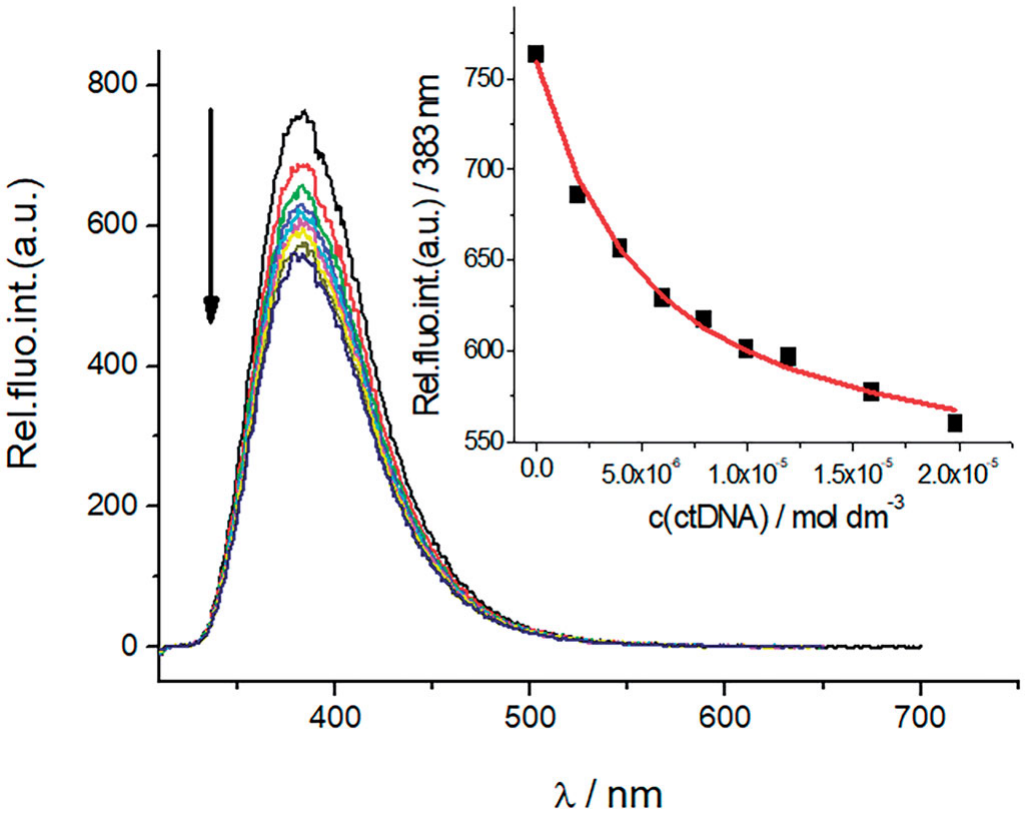
Changes in fluorescence spectrum of **14a** (*c* = 1 × 10^−6 ^M, *λ*_exc_ = 304 nm) upon titration with ctDNA (*c* = 2 × 10^−6^ to 7.8 × 10^−5 ^M); Inset: dependence of **14a** absorbance at *λ* = 385 nm on c(ctDNA), at pH = 7, sodium cacodylate buffer, *I* = 0.05 mol dm^−3^.

With the aim of investigating possible mechanisms of antiproliferative and antitrypanosomal action, we examined the DNA interactions of compounds that had exhibited the most potent submicromolar activities, **10b**, **11a**, **11b**, **12b**, **14–14c**, and **15b**. The study was performed with a double-stranded (ds-) polynucleotide, calf-thymus (ct)DNA which represents a classical B-helix consisting of 58% AT base pairs (and 42% GC base pairs). Titration with ctDNA yielded fluorescence quenching of the studied compounds (Figure 3, Table 3, Figures S19–S26, Supporting material). It can be observed that the addition of ctDNA caused a small redshift (Δ = 1–5 nm) of emission maxima.

**Table 3.** Binding constants (log*Ks*)^a^^,b^ calculated from the fluorescence titrations of benzothiazole compounds with ds-DNA at pH = 7.0 (buffer sodium cacodylate, *I* = 0.05 mol dm^−3^).

Compd.	Log*K_s_*	*I*/*I*_0_^c^
**10b**	6.6	0.12
**11a**	6.8	0.18
**11b**	6.6	0.21
**12b**	7.0	0.31
**14a**	5.6	0.54
**14b**	5.0	0.45
**14c**	-^d^	-^d^
**15b**	5.4	0.35

^a^Accuracy of *n* ± 10 – 30%, consequently log*Ks* values vary in the same order of magnitude; ^b^Processing of titration data by means of Scatchard Equation^52^ gave values of ratio *n*[bound compound]/[polynucleotide] = 0.8–0.2, for easier comparison all log *K*_s_ values were recalculated for fixed *n* = 0.6; correlation coefficients were ≥0.99 for most of calculated *K*_s_; ^c^*I*_0_ – starting fluorescence intensity of studied compounds; *I* – fluorescence intensity of compound/polynucleotide complex calculated by Scatchard equation; ^d^due to the several types of binding of compound **14c** with ctDNA, it was not possible to calculate the polynucleotide – compound complex stability constant.

The binding constants, *Ks* obtained by processing of fluorimetric titration data with the Scatchard equation^52^ are summarised in Table 3. Interestingly, the 6-amidinobenzothiazoles **10b**, **11a**, **11b**, and **12b**, which have a phenoxymethylene linker, showed higher binding affinities towards ds-DNA than compounds **14a–14c** and **15b**, which lack the linker. Non-covalent interaction of small molecules with DNA can affect the thermal stability of the double helix, which results in either an increase or decrease of the melting temperature (*T*_m_)^58^. The intercalative mode of binding usually results in an increased *T*_m_ value, while groove binding molecules can stabilise or destabilise the double-stranded structure, resulting in increased or decreased *T*_m_ values, respectively. The majority of studied compounds showed a small stabilisation effect of ctDNA (58% AT). Moreover, all 6-amidinobenzothiazoles stabilised DNA consisted of only AT sequences. The best stabilisation effects of AT-DNA were observed for **11a**, **14c**, and **15b** (Table 4).

**Table 4.** The Δ*T*_m_^a^ values (°C) of ds-DNA upon addition of ratio *r*^b^ = 0.3 of 6-amidinobenzothiazoles^c^ at pH 7.0 (sodium cacodylate buffer, *I* = 0.05 mol dm^−3^).

Compd.	ctDNA	p(dAdT)_2_
**10b**	2.7	2.3
**11a**	2.1	4.1
**11b**	1.2	1.1
**14a**	0.7	2.6
**14b**	2.2	3.6
**14c**	0	4.5
**15b**	0	5.4

^a^Difference between *T*_m_ value of free polynucleotide and complex with small molecule; error in Δ*T*_m_: ±0.5 °C; ^b^*r =* [compound]/[polynucleotide]; ^c^Changes of **12b** with increase of temperature were significant, thus Δ*T*_m_ values could not be determined.

The formation of complexes between small molecules and DNA can be monitored using circular dichroism (CD) spectroscopy. A mutual orientation of achiral small molecule and polynucleotide chiral axis, which results in an induced CD (ICD) signal, could give us additional information about modes of interaction^59^^,^^60^. Hence, the most reliable insight into modes of interaction between small molecules and DNA can be retrieved at wavelength area, *λ* > 300 nm, where ligands possess UV/Vis spectra, while DNA does not.

**Figure 4.**
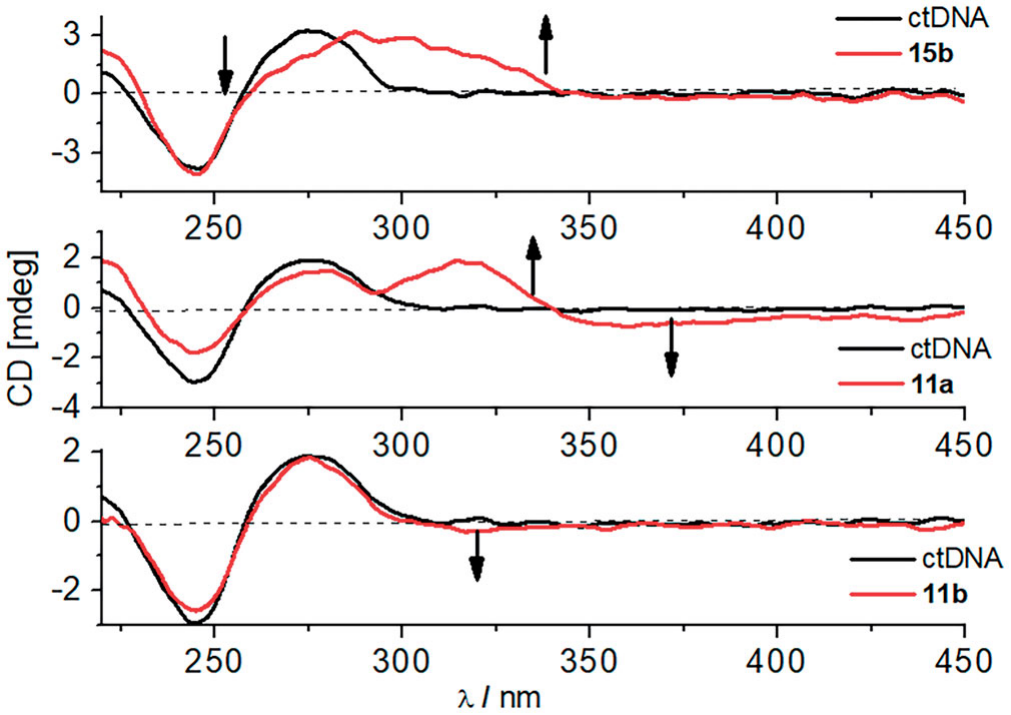
CD titration of ctDNA (*c* = 3.0 × 10^−5 ^M) with **11a**, **15b** at molar ratio, ***r*** = [compound]/[polynucleotide] = 0.5 and **11b** (molar ratio, *r*** **=** **0.1) (pH = 7.0, buffer sodium cacodylate, *I* = 0.05 mol dm^−3^).

The addition of studied compounds to ctDNA resulted in a decreased intensity of the CD band of ds-polynucleotide, ctDNA (Figure 4, Figures S31 and S32).

Furthermore, compounds **12b** and **15b** exhibited positive induced CD spectra (ICD) with ctDNA around 300 nm and 345 nm, respectively, supporting a minor groove binding mode to ctDNA^39^^,^^40^^,^^61^^,^^62^. Similar changes in CD spectra were noticed for classical minor groove binders like DAPI or Hoechst 33258^44^.

The addition of compound **11a** to a ctDNA solution resulted in the appearance of a bisignate signal with maxima at 317 and 360 nm. Such change suggests the binding of **11a** in the form of a dimer within the minor groove (Figure 4)^62^.

In the titration of ctDNA with **10b** and **11b**, a small negative ICD signal appeared, while **14a–14c** caused the small positive ICD signal upon binding to ctDNA (Figure 4, Figures S31 and S32). Such changes, whether small negative, or positive, point to an intercalative way of binding. This is additionally supported by thermal stabilisation of ctDNA and/or AT-DNA (Table 4), as well as binding constants of ∼1 µM (Table 3), which were observed with the majority of studied benzothiazoles.

The positive sign of the ICD band observed with **14a–14c** suggests that the long axis of the benzothiazole moiety is approximately perpendicular to the long axis of the basepair pocket, but still in the plane with the adjacent base pairs. The negative sign of the ICD band found for **10b** and **11b** indicates that the transition moment of the ligand is oriented “parallel” to the long axis of adjacent base pairs^42^^,^^63^.

At higher ratios, *r* ≥ 0.3, a big decrease of intensity of the CD bands and strong negative ICD spectra observed with **14a–14c** can be attributed to non-specific aggregation of non-intercalated molecules along the DNA backbone, possibly within major grooves^43^.

## Conclusions

The 6-amidinobenzothiazoles **10a–10d**, **11a–11d**, **12a–12d**, **13a–13d**, **14a–14d**, and **15a–15d**, containing distributed highly hydrophilic cationic moieties and hydrophobic aromatic components were designed and synthesised with the aim of performing antiproliferative and antiprotozoal evaluations. The antiproliferative assessment showed that imidazoline moieties improved the growth-inhibitory effects of **11a–11d** and **14a–14d** and that benzothiazole directly connected to the 1,2,3-triazole in **14a–14d** additionally increased inhibitory activity. Thus, benzothiazole imidazolines **11a**, **14a** and **14c** showed the most pronounced inhibitory effects on all the tumour cell lines studied (**11a**: IC_50_ = 0.49 µM, MCF-7; **14a**: IC_50_ = 0.25 µM, SW620, IC_50_ = 0.45 µM, CFPAC-1; **14c**: IC_50_ = 0.38 µM, HeLa). However, 6-amidinobenzothiazoles also affected the proliferation of the non-tumour HFF-1 cell line.

Similarly, the antitrypanosomal evaluations showed that benzothiazole imidazolines **11a–11c** and **14a–14c** exhibited the best potency, with values that paralleled antiproliferative activity. In contrast to antitumor results, a direct fusion of benzothiazole to 1,2,3-triazole decreased antitrypanosomal activity. 6-Imidazolinobenzothiazole **11b** containing *p*-chlorophenyl at N-1 of 1,2,3-triazole displayed the highest antitrypanosomal potency (IC_50_ = 0.09 µM, IC_90_ = 0.12 µM). UV–Vis and CD spectroscopy, as well as thermal denaturation assays, indicated the binding affinities of 6-amidinobenzothiazoles towards ctDNA. Strong positive ICD bands supported minor groove binding, as the dominant binding mode of **11a**, **12b** and **15b**, while small negative and positive ICD signals identified intercalation, as the predominant binding mode of **10b** and **11b** and **14a–14c**.

We may conclude that the 6-amidinobenzothiazoles, which exhibit antiproliferative and antitrypanosomal potency in the submicromolar range and DNA interacting properties, warrant further structural optimisation to reduce their toxicity against normal cells, with the aim of developing novel antitumor or/and anti-HAT agents.
